# The gestational diabetes future diabetes prevention study (GODDESS): A partially randomised feasibility controlled trial

**DOI:** 10.1371/journal.pone.0273992

**Published:** 2022-12-30

**Authors:** Judith Parsons, Rita Forde, Anna Brackenridge, Katharine F. Hunt, Khalida Ismail, Trevor Murrells, Anna Reid, Helen Rogers, Rebecca Rogers, Angus Forbes

**Affiliations:** 1 Care in Long Term Conditions Research Division, Florence Nightingale Faculty of Nursing, Midwifery and Palliative Care, King’s College London, London, United Kingdom; 2 Guy’s and St Thomas’ NHS Foundation Trust, London, United Kingdom; 3 Diabetes & Nutritional Sciences Division, Diabetes Research Group, King’s College London, London, United Kingdom; 4 Department of Psychological Medicine, Institute of Psychiatry, King’s College London, London, United Kingdom; 5 Methodologies Research Division, Florence Nightingale Faculty of Nursing, Midwifery and Palliative Care, King’s College London, London, United Kingdom; Public Library of Science, UNITED STATES

## Abstract

**Objectives:**

To assess the feasibility of an ante- and post-natal lifestyle intervention for women with gestational diabetes mellitus (GDM) to reduce type 2 diabetes risk.

**Design:**

A partially randomised patient preference feasibility trial.

**Setting:**

Diabetes antenatal clinics in two inner-London hospitals, UK.

**Participants:**

Pregnant women ≥18 years with a GDM diagnosis and pre-pregnancy body mass index of ≥25kg/m^2^.

**Intervention:**

Participants in the intervention group were offered four motivational interview-based sessions (two antenatally and two postnatally, at 3 and 6 months postpartum), a WhatsApp support group, a FitBit and electronic self-help resources.

**Outcome measures:**

Recruitment; retention; intervention dose received; data completion; adaptions; proportion achieving ≥5% weight loss; weight change, blood glucose; blood pressure; diet, physical activity, breastfeeding and depression. Clinical outcomes were measured at baseline and 6 months postpartum.

**Results:**

50 participants were recruited from 155 eligible women (32% recruitment rate). Thirty-four were recruited to the intervention group (23 following randomisation (RI-group) and 11 based on preference (PI-group)); and 16 to the control group (13 randomised (RC-group) and 3 preference (PC-group)). Attrition was 44% (n = 22/50). Forty-six percent (n = 6) of the intervention group (25% (n = 2) of the RI-group and 80% (n = 4) of the PI-group) achieved ≥5% weight loss compared to 8% (n = 1) in the control group (95% confidence interval (CI) -0.69 to 0.07). Mean weight change was -2.1kg±9.0 in the intervention group (0kg±5.4 in the RI-group and -5.4kg±13.0 in the PI-group) compared to +4.4kg±4.9 in the control group (RC +4.4kg ±5.3 and PC +4.7kg ±3.1, 95% CI -12.4 to 0.2).

**Conclusions:**

Recruitment was feasible, but strategies to improve retention are needed. The findings suggest the intervention can support women with GDM to lose weight. The observed weight loss was primarily in women who preferred the intervention. Therefore, future trials may need to adopt a preference design and consider factors associated with preference.

**Trial registration:**

**Trial registration:** ISRCTN52675820 https://www.isrctn.com/ISRCTN52675820?q=ISRCTN52675820&filters=&sort=&offset=1&totalResults=1&page=1&pageSize=10&searchType=basic-search.

## Introduction

Gestational diabetes mellitus (GDM) is a common condition, occurring in around 10% of pregnancies globally [1], and is increasing [2]. GDM is a state of hyperglycaemia that occurs when a woman’s endogenous insulin production is unable to compensate for the hormonal changes that occur in pregnancy, making her more insulin resistant. Many of the risk factors for GDM are common to those for the development of type 2 diabetes mellitus (T2DM), in particular obesity and being of Black African, Black Caribbean or South Asian descent [3]. The risk of women with GDM developing future T2DM is ten times that of women with normoglycaemic pregnancies [4] and around 50% of women with GDM will go on to develop T2DM [5]. In addition, women with GDM have a 36% chance of having GDM in a subsequent pregnancy [6], and the children of women with GDM are more likely to become obese or develop T2DM in adulthood [7]. GDM is associated with adverse fetal, infant and maternal pregnancy outcomes [8], and T2DM increases the risk of future morbidity and premature mortality [9]. While women with GDM are a high-risk population for developing T2DM, they are in frequent contact with health services, providing an opportunity to identify them and intervene.

A substantial proportion of cases of T2DM can be delayed or prevented through diet, physical activity and weight loss. While previous large scale studies of lifestyle interventions in high-risk populations have shown significant reductions in the incidence of T2DM, ranging from 29% to 67% [10–12], definitive research on the prevention of T2DM in women exposed to GDM is limited. A sub-analysis of data from the Diabetes Prevention Program showed that women with previous GDM (mean 12 years prior to the study) experienced a 50% reduction in incidence of T2DM after lifestyle intervention [13]. More recently several randomised controlled trials (RCTs) exploring the impact of lifestyle interventions on diabetes risk, behavioural and metabolic markers after GDM have been undertaken. A meta-analysis of eight studies investigating the incidence of diabetes following lifestyle interventions showed a 25% (RR = 0.75; 95% confidence interval (CI): 0.55–1.03) reduction in diabetes risk. This reduction was greater when the intervention was initiated less than 6 months after the birth (RR = 0.61; 95%CI: 0.40–0.94) [14]. Overall, studies suggest T2DM can be prevented in women with GDM. However, the lifestyle interventions included in this analysis varied greatly in terms of content, delivery and context and none were UK-based.

Therefore, we used learning from previous studies to inform the development of a theoretically modelled lifestyle intervention for women with GDM incorporating motivational interviewing (MI) and current best-practice guidance for diabetes prevention [15]. Following the first phases of the UK Medical Research Council guidance for developing complex interventions [16] we conducted a meta synthesis of previous studies [17] as well as a qualitative study to explore how best to adapt and deliver a lifestyle diabetes prevention intervention for women exposed to GDM. An important finding of this work was that GDM can be very emotionally distressing for women, further complicating their interest in changing their lifestyle behaviours. We synthesised these findings to formulate a logic model for the intervention that identified the following active ingredients to support lifestyle change: addressing emotional needs; focussing on the woman as well as their infant; conveying personalised risk in a meaningful way; and interaction with peers. The logic model informed the development of a multimodal intervention, The GestatiOnal Diabetes future DiabEteS prevention Study (GODDESS), which aims to reduce the risk of T2DM in women with GDM primarily by enabling weight loss through the setting or personalised lifestyle goals using motivational interviewing. In this paper we report the findings of a feasibility study to estimate the utility and acceptability of the intervention and its effect, to inform how best to optimise the intervention and design a definitive trial.

## Methods

The study was a feasibility trial with an integrated process evaluation following the MRC Process Evaluations guidelines [18]. The aim of the study was to consider the feasibility of the intervention and the study design (recruitment, randomisation, retention, and data collection processes). The objectives were to:

- Examine the feasibility of conducting a trial of a lifestyle intervention with women with GDM, including assessing the feasibility of the trial parameters.
- Identify the proportion achieving a weight change ≥5% from baseline to 6 months postpartum (the primary effectiveness outcome) to inform a sample size calculation for a large-scale definitive trial.

### Design and setting

This feasibility study used a partially randomised controlled trial design. Participants were initially randomised to either the intervention or control conditions. Women who refused randomisation were allowed to express a preference for either condition. Including the women who refused randomisation enabled us to explore how preference might mediate or confound outcomes [19–21]. In the context of this feasibility study, allowing preference also provided the opportunity to consider whether preference should be considered as a basis for the design of a future study. Analyses were performed as: 1) an on-treatment analysis of intervention and control groups (including those with a preference), and 2) on treatment analysis exclusive to those randomised to the groups.

### Participants

Eligibility criteria were: current diagnosis of GDM (based on the UK National Institute of Health Excellence 2015 criteria (fasting venous plasma glucose ≥5.6 mmol/L or 2 hour 75g glucose load venous plasma glucose ≥7.8 mmol/L) or HbA1c at booking appointment ≥6% / 42 mmol/mol); body mass index (BMI) ≥25 kg/m^2^ at pregnancy booking appointment (8–12 weeks gestation); <35 weeks gestation; ≥18 years of age (the gestational age inclusion criteria was amended from the protocol to reflect the current clinic population and allow more women to participate). Potential participants were deemed ineligible if they were unable to consent, had severe mental illness or did not speak English. Participants were recruited opportunistically from antenatal diabetes clinics in two inner London hospitals between October 2018 and May 2019 and follow up data for the last participant was collected in March 2020.

### Consent

Eligible participants were identified by a diabetes health professional during routine antenatal visits. Potential participants were provided with verbal and written information, and interested women were contacted within 48 hours by a researcher to discuss the study further and subsequently to arrange written consent. Participants were then given a baseline questionnaire to complete, after which they were randomised to either the control or intervention group. If women stated a strong preference, they were then permitted to change to the other group to support participation in the study. Preferences were considered in the data analysis.

### Randomisation and blinding

Block randomisation, stratified by hospital site, was conducted by a statistician not involved in the study using a computer-generated sequence, and the allocations were placed in sealed envelopes by another external person so that the allocation was fully blinded. Given the nature of the intervention, it was not possible to blind allocation of treatment from participants, researchers or the intervention facilitators.

### Intervention and control conditions

**Fig 1:**
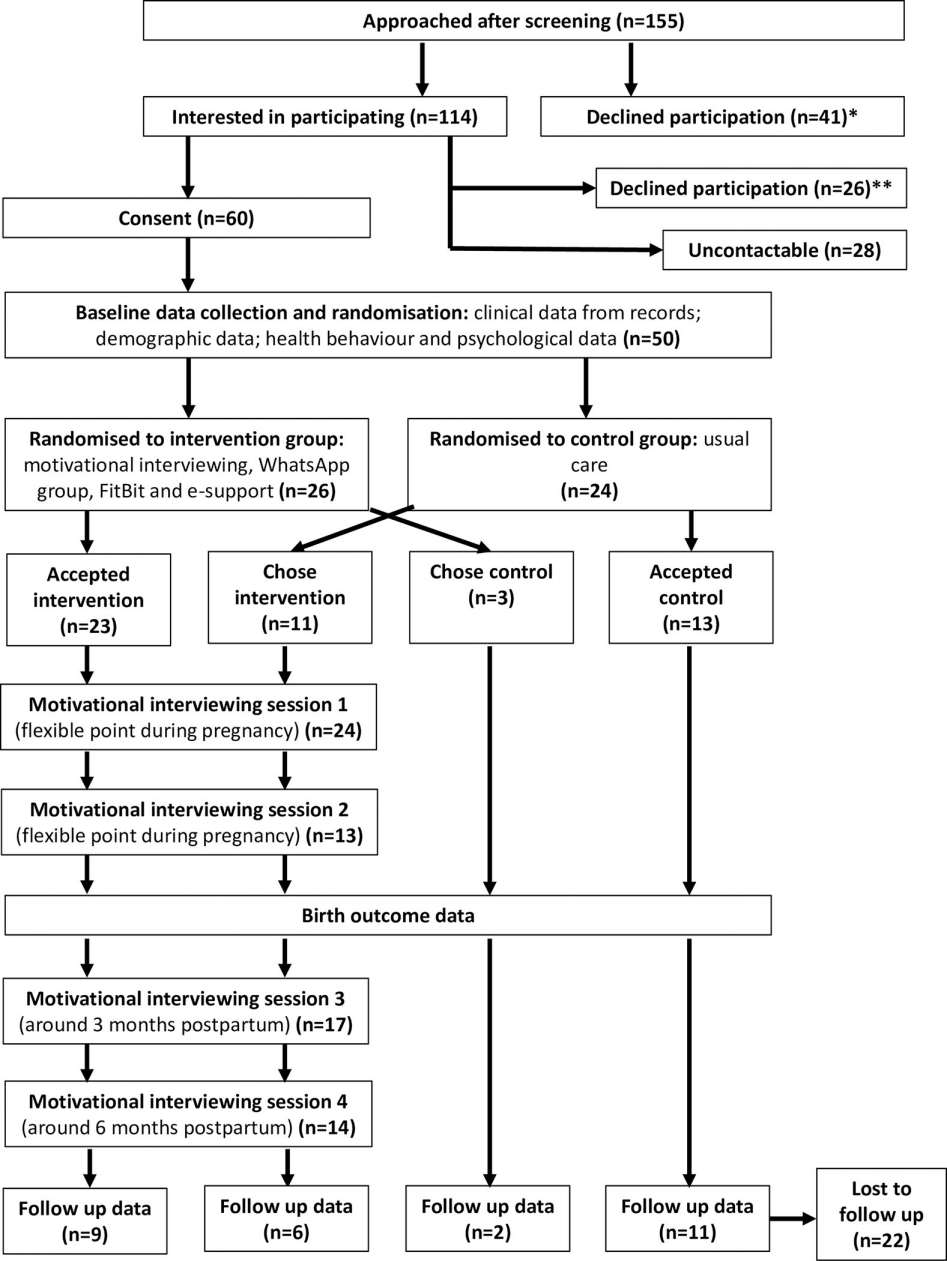
Overview of GODDESS model (underpinning techniques and behavioural mechanisms). EPE = ‘Elicit, Provide, Elicit’. OARS = ‘Open questions, Affirmations, Reflections, Summaries’. EE = ‘Express Empathy’. RR = ‘Roll Resistance’. DD = ‘Develop Discrepancy’. SS = ‘Support Self-efficacy’. SMART = Simple, Measurable, Achievable, Relevant, Time based.

The intervention was informed by current best evidence for diabetes prevention programmes using the IMAGE (Development and *Im*plementation of *a* European *G*uidelin*e* and Training Standards for Diabetes Prevention) toolkit [15]. The lifestyle targets and behaviour change model within the toolkit were adapted to the needs of the GDM population and current guidance on dietary management and exercise in pregnancy [22]. The aim of the intervention was to facilitate lifestyle change to achieve a 5% reduction in pre-pregnancy body weight. Following the IMAGE model, the GODDESS intervention is underpinned by the theory of planned behaviour [23] and is delivered using MI techniques, as outlined in Fig 1. MI was chosen as it has a good fit with the underlying approach and has been shown to be effective in supporting weight loss in those who are overweight [24]. Intervention content and delivery was also informed by the prior qualitative work and patient and public involvement (PPI) group, which indicated the need for emotional support within the behavioural intervention. PPI also suggested that an intervention initiated in pregnancy and continuing postpartum was optimal, and therefore we offered two antenatal (timing dependent on timing of diagnosis and recruitment) and two postpartum sessions (at three and again at six months after the birth). The intervention sessions were delivered by one of two diabetes specialist nurses; both were trained and experienced in MI techniques. Following the IMAGE model of behaviour change, SMART (specific, measurable, achievable, relevant, time-based) goal-setting was incorporated into each session. Sessions were piloted with women with recent GDM, and the two facilitators met frequently during the study to compare techniques and discuss any issues arising. Sessions were offered to participants in diabetes out-patient clinic rooms in the participating hospitals and lasted up to one hour. At each follow-up session the goals from the previous session were reviewed and new goals set. The sessions were supplemented by: a WhatsApp group to enable peer support; text reminders based on personal SMART goals; a FitBit for the participant to monitor activity levels; and a website with information on reducing the risk of T2DM. No financial incentive was provided to participants, but those in the intervention group were permitted to keep the FitBit.

Women in the control arm received usual care in the diabetes pregnancy clinic (typically fortnightly outpatient appointments with diabetes nurses and doctors to optimise glycaemic control and dieticians to advise on diet and lifestyle if necessary). In addition, after having clinical follow up data collected, participants were sent an email or text message with an explanation of the result and referred to their GP if their glycated haemoglobin (HbA1c) was ≥6.5%. At the end of the study, they were offered the opportunity to access a support session.

### Study outcomes

The study outcomes were identified to explore the feasibility of the intervention and to inform the design of a future trial [18]. Specific feasibility outcomes were:

- The proportion of women screened, consented and randomised, and reasons for non-participation
- The proportion of data completion at baseline and follow up, withdrawal from the intervention and/or follow up and reasons for withdrawal
- The amount of the intervention that participants received (measured by number of MI sessions attended and number of WhatsApp messages sent)
- Intervention fidelity and any adaptations to the intervention

The primary effectiveness outcome was the proportion of women achieving ≥5% weight loss between pregnancy booking and follow-up. Weight loss has been shown to be one of the main predictors in reducing diabetes risk, with analysis of the Diabetes Prevention Program data showing that for every kilogram of weight lost, diabetes risk reduces by 16% [25]. Penn et al. [26] showed 65% reduction in the risk of developing T2DM in women who lost 5% of their pregnancy booking weight 12 months after a GDM pregnancy. The secondary effectiveness outcomes were: weight change (kg); HbA1c; blood pressure; diet (fat and calorie intake); level of physical activity; breastfeeding uptake and depression. All these measures have been independently associated with future T2DM risk [27–31].

### Data collection

Baseline weight, taken at <12 weeks’ gestation, was collected from the pregnancy booking record. Studies have shown that pregnancy booking weight, taken in the first trimester, is equivalent or a near match to pre-pregnancy weight [32] and is more reliable than self-reported pre-pregnancy weight [33]. Baseline HbA1c, recorded at GDM diagnosis, was also taken from medical records. Follow-up weight and HbA1c samples were collected by clinic nurses or healthcare assistants not connected to the study at six months postpartum.

Diet was assessed through online repeated 24-hour recall (three repeats requested on different days of the same week, with a mean average value used) using a validated online tool–Intake24 [34]. Participants were given the choice to complete this either with a researcher or in their own time. Physical activity was assessed through the general practice physical activity questionnaire [35]. The GPPAQ is a validated screening tool used in primary care to assess the physical activity of adults. The tool asks two questions and then categorises participants into active, moderately active, moderately inactive, and inactive. Although GPPAQ was developed as a screening method, it has been used in trials to assess change in activity [36,37].

Depression was assessed using the Edinburgh Postnatal Depression Scale (EPDS) [38] and Patient Health Questionnaire 9 (PHQ9) [39], which are the tools recommended by the National Institute of Clinical Excellence (NICE) to detect ante- and postnatal depression in the UK. The EPDS is a 10-question screening tool that has been found to have high sensitivity [38,40] both in pregnancy and the postpartum period. The cut-off of 13 out of a maximum of 30 was used to indicate probable depression. PHQ9 is a 9-question validated screening tool for depression severity and has been found to be sensitive to mood changes in both pregnant [41] and postpartum [42] women. We used a PHQ9 score of >4 to indicate clinically significant depressive symptoms (mild to severe). If screening indicated that a participant had moderate to severe depression based on the PHQ9, or a participant had answered anything other than ‘never’ to questions concerning thoughts of self-harm in either the PHQ9 or the EPDS, they were contacted immediately and offered a referral to professional support.

Infant feeding (breast and/or formula) at birth and follow up was also recorded in the questionnaire. Age and gestational age were taken from hospital records. Other demographic data were obtained through the questionnaire including employment, education, postcode, parity and incidences of GDM. Postcodes were matched to the UK Index of Multiple Deprivation (IMD) and analysed in quintiles. Birth outcome data (gestational age and infant weight) were also collected from birth records. Reasons for declining participation, withdrawal from the intervention and non-completion of follow-up data, as well as any further participant feedback were recorded.

### Sample size

The sample was determined following current consensus recommendations for feasibility studies which is for 12–25 participants in each study arm [43–46]. We aimed to recruit 60 participants to remain concordant with these recommendations, allow for potential attrition and for some estimation of the effect size and standard deviation of effect in the primary effectiveness outcome (proportion of women achieving ≥5% weight loss) to inform the calculation of sample size for a future trial.

### Ethics and trial registration

The study was approved by the UK Research Ethics Committee (reference 13/SW/0141). The study was registered with ISRCRN (trial number ISRCTN52675820).

### Patient and public involvement

Preliminary interviews and focus groups were conducted with 50 women with recent GDM in order to help develop the intervention [47,48]. Some participants formed a PPI group, which fed into the development of the intervention and study design, provided feedback on data collection methods and tested out elements of the intervention.

### Data analysis

Baseline characteristics of intervention and control groups were compared, with Chi-square tests for categorical data and independent samples t-tests for continuous variables. The number of women losing ≥5% of body weight was cross tabulated by group. One way ANCOVA (analysis of covariance) was used to estimate the difference in continuous variables (weight, blood pressure etc.) between intervention and control group at follow-up (six months) not adjusting and then adjusting for baseline values to provide estimates of effect with 95% confidence intervals. Odds ratios were calculated according to Altman’s methodology [49]. Data was checked for normality using a Shapiro-Wilk test before testing. The primary analysis was performed as an on-treatment analysis due to the impact of preference on group allocations. Participants lost to follow-up were excluded from the analysis. Analysis was conducted using SPSS (version 26).

## Results

### Primary feasibility outcomes

#### Recruitment and randomisation

**Fig 2:**
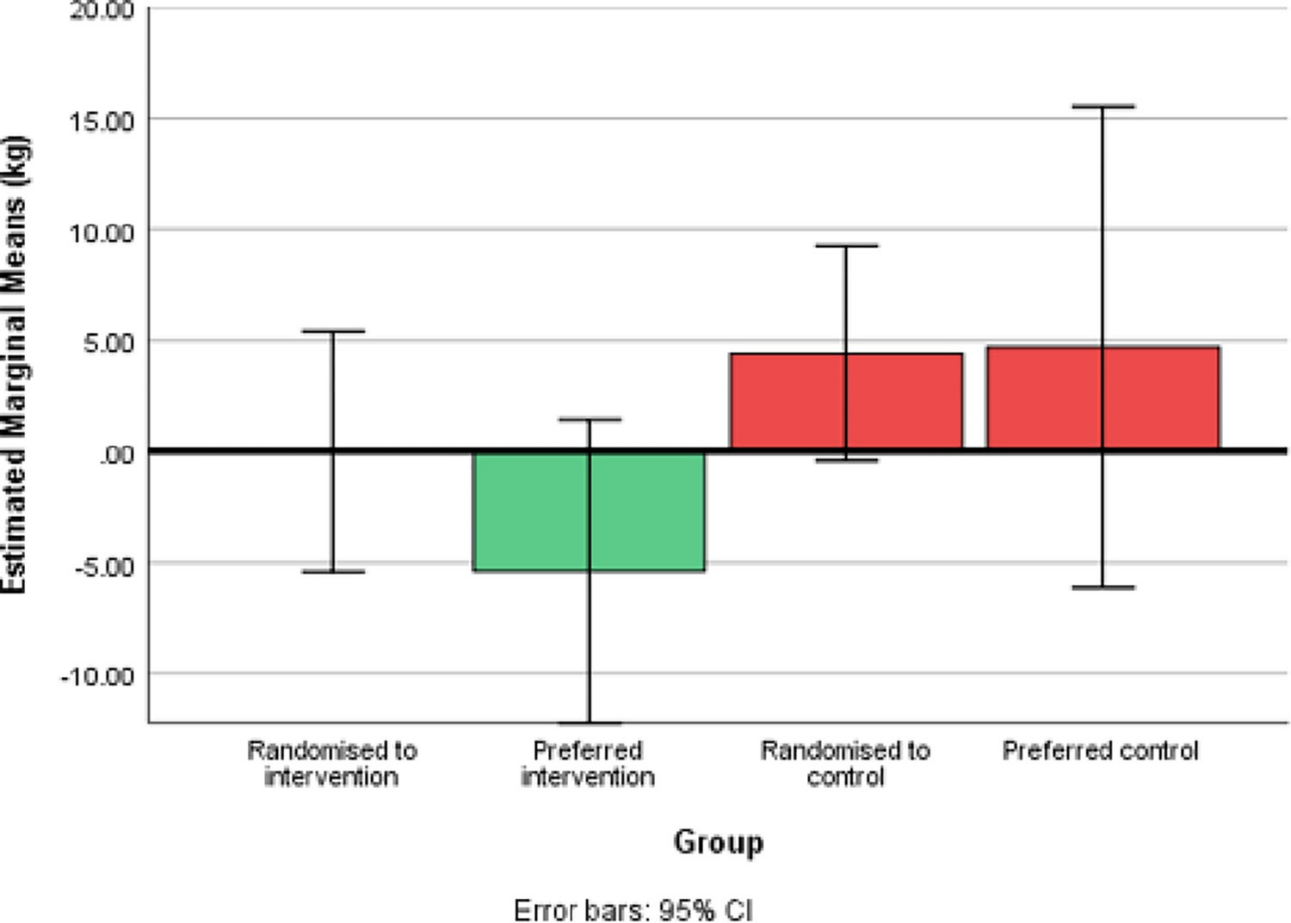
Study flow chart. *Reasons for declining participation. No reason given n = 12. English not good enough n = 8. Too busy n = 5. Do not believe they have GDM n = 4. Not interested in research n = 2. Distance n = 2. Lack of childcare n = 2. Study too arduous or long n = 2. Already doing other research n = 2. Believes GDM will resolve once baby is born n = 1. Had GDM before so knows what she needs to do n = 1. **Reasons for declining participation after initial interest. Too busy n = 5. No reason given n = 4. Health issue (mother or baby) n = 3. Distance / travel is too much n = 3. Difficult circumstances n = 3. Involved in other research n = 2. Lack of childcare n = 2. Doesn’t speak good enough English n = 1. Too many appointments already n = 1. Fatigue n = 1. Believes GDM will resolve n = 1.

Study recruitment took just over six months to complete. A flow chart of the study is shown in Fig 2. During the recruitment period 155 eligible patients were approached, of which 39% (n = 60) consented, 27% (n = 41) declined participation on first contact, 17% (n = 26) declined participation on further contact, and 18% (n = 28) were subsequently uncontactable. Reasons for not taking part in the study were collected for 58% (n = 55) of the 95 women who did not consent. Reasons included: a lack of confidence in their ability to speak or understand English; a lack of time; disbelief in their GDM diagnosis; travel constraints; and difficult circumstances such as illness or a lack of childcare.

During the recruitment period 213 and 324 women with GDM were under the care of hospitals 1 and 2 respectively (total n = 537). It was not logistically possible to screen all patients for eligibility and no data on language spoken nor mental illness were available. However, an estimated 402 of these women (estimated from BMI data that was only available for 53%) would have met the study’s BMI criteria. Based on BMI, this would indicate that the sample recruited constituted approximately 15% of the total estimated eligible population (n = 402).

Of the 60 participants who consented, four participants withdrew and six were lost before randomisation, leaving 50 women who were randomised: 26 to the intervention group and 24 to the control group. Eleven women randomised to the control group and three women randomised to the intervention group stated a strong preference for the other group, which resulted in 34 participants in the intervention group (23 randomised to intervention (IR-group) and 11 who preferred the intervention (IP-group)) and 16 in the control group (13 randomised to control (CR-group) and 3 who preferred control (CP-group)); meaning that 72% (n = 36) of participants were willing to be randomised (see Fig 2).

#### Participant characteristics

Participants had a mean gestational age of 28.1 (±5.3) weeks at recruitment. At pregnancy booking, mean BMI was 34 kg/m^2^ (±5.6) and mean weight was 92kg (±16.3). Mean age was 35 (±4.6) years and 58% (n = 29) already had one or more child. In terms of ethnicity 40% (n = 19) were Black, 27% (n = 13) White, 19% (n = 9) Asian, 8% (n = 4) other and 6% (n = 3) were from Mixed ethnicity backgrounds. Sixty-eight per cent (n = 25) had received university-level education and 62% (n = 31) were employed or self-employed. Nearly half (46%, n = 23) lived in local authority wards that were in the two most socially deprived quintiles. Mean HbA1c at diagnosis of GDM was 5.7% (±0.4), calorie intake 1334.8 kcal (±471.5), fat intake 55.5 grams (±28.0), systolic blood pressure was 117.2 mmHg (±14.2) and diastolic 72.8 mmHg (±10.6), and 60% (n = 24) were either physically active or moderately physically active. Overall, intervention and control groups were comparable at baseline (see Table 1). The randomised only groups were also comparable, other than fat intake, which was higher in the intervention group (61.2 grams (±28.8) compared to 37.6 grams (±15.2), p = 0.02).

**Table 1:** Baseline and birth outcome data by group: All participants and randomised participants.

Variable	All participants by group			Randomised participants		
	Control (n = 16)	Intervention (n = 34)	P*	Control (n = 13)	Intervention (n = 23)	P*
	Mean ±SD or n (%)			Mean ±SD or n (%)		
Gestational age at recruitment *(weeks)*	29.6 ±2.6	27.4 ±6.1	0.82	30.1±2.3	27.9±4.8	0.13
Age at recruitment *(years)*	34.5 ±5.9	35.4 ±4.0	0.59	34.0±6.0	35.4±4.7	0.49
Primapara *(number)*	6 (37.5%)	15 (44.1%)	0.76	4 (33.3%)	9 (40.9%)	0.73
First incidence of GDM	1.4 ±0.7	1.3 ±0.5	0.57	7 (70.0%)	15 (75.0%)	1.0
Ethnic group			0.23			0.19
*Black / African / Caribbean / Black British*	6 (40.0%)	13 (39.4%)		4 (33.3%)	9 (40.9%)	
*White British or other White*	4 (26.7%)	9 (27.3%)		3 (25.0%)	8 (36.4%)	
*Asian*	5 (33.3%)	4 (12.1%)		5 (41.7%)	2 (9.1%)	
*Other ethnic group*	0 (0.0%)	4 (12.1%)		0 (0.0%)	1 (4.5%)	
*Mixed*	0 (0.0%)	3 (9.1%)		0 (0.0%)	2 (9.1%)	
Education level			0.50			0.52
*Degree*	8 (72.7%)	17 (65.4%)		7 (77.8%)	11 (61.1%)	
*Further education*	3 (27.3%)	6 (23.1%)		2 (22.2%)	5 (27.8%)	
*GSCE or equivalent*	0 (0%)	3 (11.5%)		0 (0.0%)	2 (11.1%)	
Index of Multiple Deprivation quintile grouped			0.67			0.83
*1–2 (most deprived)*	6 (54.5%)	17 (58.6%)		6 (60.0%)	14 (70.0%)	
*3*	4 (36.4%)	7 (24.1%)		3 (30.0%)	4 (20.0%)	
*4–5 (least deprived)*	1 (9.1%)	5 (17.2%)		1 (10.0%)	2 (10.0%)	
Employment status			0.48			0.84
*Employed or self-employed*	9 (69.2%)	22 (78.6%)		8 (72.7%)	15 (78.9%)	
*Unemployed*	3 (23.1%)	2 (7.1%)		2 (18.2%)	2 (10.5%)	
*Homemaker*	1 (7.7%)	3 (10.7%)		1 (9.1%)	2 (10.5%)	
*Student*	0 (0.0%)	1 (3.6%)		0 (0.0%)	0 (0.0%)	
Gestational age at diagnosis *(weeks)*	23.1 ±7.4	21.2 ±7.6	0.41	24.1±7.2	21.8±6.8	0.37
BMI at booking *(kg/m*^*2*^*)*	33.1 ±4.5	34.6 ±6.1	0.37	32.6±4.1	36.1±6.4	0.09
Weight at booking *(kg)*	89.4 ±14.4	92.8 ±17.2	0.51	87.7±13.2	97.0±17.6	0.11
HbA1c at GDM diagnosis *(%)*	5.7 ±0.4	5.8 ±0.4	0.41	5.6±0.4	5.7±0.4	0.50
Systolic blood pressure at diagnosis (mmHg)	118.0 ±15.67	116.8±13.9	0.83	115.0±15.3	118.1±14.4	0.63
Diastolic blood pressure at diagnosis (mmHg)	73.7 ±7.5	72.5 ±11.8	0.76	72.4±7.4	75.4±10.9	0.48
Daily calorie intake (kcal)	1125.8 ±449.6	1422.8 ±463.6	0.14	1006.4±320.8	1440.2±534.9	0.07
Daily fat intake (grams)	47.5 ±31.4	58.9 ±26.5	0.34	37.6±15.2	61.2±28.8	**0.02**
Physically active or moderately active	6 (50.0%)	18 (64.3%)	0.49	5 (50.0%)	11 (57.9%)	0.49
Probable depression (EPDS)	4 (30.8%)	9 (34.6%)	1.00	4 (36.4%)	4 (22.2%)	0.34
Any depression (PHQ9)	4 (30.8%)	16 (64.0%)	0.09	4 (36.4%)	8 (47.1%)	0.44
**Birth outcome data**						
Birth weight of baby (kg)	3.2 ±0.4	3.3 ±0.3	0.52	3.2±0.4	3.3±0.3	0.58
Gestational age at birth (weeks)	38.4 ±1.4	38.0 ±1.4	0.34	38.4±1.3	38.0±1.3	0.35

* p values for categorical variables were calculated with Chi square test, and for continuous variables with independent samples t-test.

#### Participant retention and data completion

Of the 50 participants, 22 were lost to follow up during the study, resulting in 28 participants (56% of those randomised) completing the study (completing the study was defined as providing at least clinical (weight, HbA1c and blood pressure) or questionnaire follow up data). Reasons for withdrawing from the study were given in some cases (n = 7), and contact was lost with 15 women without them formally withdrawing. One intervention participant provided follow up data without completing the intervention due to covid-19 restrictions. Reasons for withdrawing during pregnancy included: belief GDM had gone, being too busy, questionnaire was too personal, or no incentive. For those withdrawing after the birth, reasons included moving away, not wanting to travel to the hospital, spending extended time abroad, no longer having GDM and difficult family situations. Three women were lost to follow up because data collection was halted prematurely by the Covid-19 pandemic restrictions and a further woman did not provide clinical data for the same reason.

Participants in the control group were more likely to complete the study than those in the intervention group. Those who completed were more likely to have attended university than not, and more likely to have been diagnosed with GDM at a later gestation than those who did not complete. Preference was not associated with any difference in completion (see Table 2).

**Table 2:** Baseline and birth outcome data by completers and non-completers.

Variable	Completers (n = 28) Mean ±SD or n (%)	Non completers (n = 22) Mean ±SD or n (%)	P*
Gestational age at recruitment *(weeks)*	29.4 (4.0)	26.4 (6.3)	0.06
Age at recruitment *(years)*	34.5 (4.4)	36.0 (5.0)	0.29
Primapara *(number)*	15 (53.6%)	6 (27.3%)	0.09
Incidences of GDM (including this one)	1.22±0.5	1.50 ±0.5	0.09
Ethnic group			0.78
*Black / African / Caribbean / Black British*	10 (37.0%)	9 (42.9%)	
*White British or other White*	7 (25.9%)	6 (28.6%)	
*Asian*	6 (22.2%)	3 (14.3%)	
*Other ethnic group*	3 (11.1%)	1 (4.8%)	
*Mixed*	1 (3.7%)	2 (9.5%)	
Education level			**0.05**
*Degree*	17 (85.0%)	8 (47.1%)	
*Further education*	2 (10.0%)	7 (41.2%)	
*GSCE or equivalent*	1 (5.0%)	2 (11.8%)	
Index of Multiple Deprivation quintile grouped			0.38
*1–2 (most deprived)*	12 (52.2%)	11 (64.7%)	
*3*	6 (26.1%)	5 (29.4%)	
*4–5 (least deprived)*	5 (21.7%)	1 (5.9%)	
Employment status			0.13
*Employed or self-employed*	21 (87.5%)	10 (58.8%)	
*Unemployed*	2 (8.3%)	2 (11.8%)	
*Homemaker*	0 (0.0%)	1 (5.9%)	
*Student*	1 (4.2%)	4 (23.5%)	
Gestational age at diagnosis *(weeks)*	24.1 ±6.2	18.8 ±8.1	**0.02**
BMI at booking *(kg/m*^*2*^*)*	33.1 ±4.8	35.4 ±6.5	0.16
Weight at booking *(kg)*	88.8 ±14.9	95.3 ±17.6	0.17
HbA1c at GDM diagnosis *(%)*	5.7 ±0.4	5.8 ±0.4	0.69
Systolic blood pressure at diagnosis (mmHg)	113.9 ±12.1	121.1 ±15.9	0.14
Diastolic blood pressure at diagnosis (mmHg)	71.9 ±7.6	73.9 ±13.5	0.60
Daily calorie intake (kcal)	1360.3 ±429.1	1302.8 ±537.7	0.76
Daily fat intake (grams)	61.7 ±29.4	47.8 ±25.2	0.21
Physically active or moderately active	15 (65.2%)	9 (52.9%)	0.52
Probable depression (EPDS)	9 (40.9%)	4 (23.5%)	0.32
Any depression (PHQ9)	11 (52.4%)	9 (52.9%)	1.00
Group			**0.02**
*Intervention*	15 (53.6%)	19 (86.4%)	
*Control*	13 (46.4%)	3 (13.6%)	
Randomised or preference			0.59
*Randomised*	20 (55.6%)	16 (44.4%)	
*Preference*	8 (57.1%)	6 (42.9%)	
**Birth outcome data**			
Birth weight of baby (kg)	3.2 ±0.4	3.3 ±0.4	0.21
Gestational age at birth (weeks)	38.3 ±1.5	37.8 ±1.0	0.27

* p values were calculated from Chi-square tests for categorical data and independent samples t-tests for continuous variables.

Data completeness for each variable is outlined in Table 3. Data collection was feasible but some participants reported that questionnaires were too long, emotionally difficult or repetitive and questioned the validity of the diet questionnaire (data from research log book).

**Table 3:** Data completion at baseline and follow up.

Variable	Completed data (number (%))	
	Control (n = 13)	Intervention (n = 15)
**Baseline**		
Gestational age at recruitment	13 (100%)*	15 (100%)*
Weight	13 (100%)*	15 (100%)*
HbA1c	13 (100%)*	15 (100%)*
BMI	13 (100%)*	15 (100%)*
Birth weight of baby	13 (100%)*	14 (93%)*
Gestational age at birth	13 (100%)*	14 (93%)*
Ethnic group	12 (92%)^	15 (100%)^
Gestational age at diagnosis	12 (92%)*	15 (100%)*
Age at recruitment	10 (77%)*	14 (93%)*
Employment status	10 (77%)^	14 (93%)^
Number of children	10 (77%)^	14 (93%)^
Incidences of GDM	10 (77%)^	14 (93%)^
Physical activity	10 (77%)^	14 (93%)^
Depression (EPDS)	10 (77%)^	13 (87%)^
Depression (PHQ9)	10 (77%)^	12 (80%)^
Index of Multiple Deprivation	9 (69%)^	14 (93%)^
Blood pressure	9 (69%)*	9 (60%)*
Education level	8 (62%)^	13 (87%)^
Calorie intake	7 (54%)~	9 (60%)~
Fat intake	7 (54%)~	9 (60%)~
**Follow up**		
Weight	12 (92%)^+^	13 (87%)^+^
HbA1c	12 (92%)^+^	13 (87%)^+^
Physical activity	12 (92%)^	11 (73%)^
Depression (EPDS)	12 (92%)^	11 (73%)^
Depression (PHQ9)	12 (92%)^	11 (73%)^
Breast, formula or mixed feeding at birth	12 (92%)^	11 (73%)^
Breast, formula or mixed feeding at follow up	11 (75%)^	10 (67%)^
Blood pressure	11 (69%)^+^	12 (80%)^+^
Calorie intake	4 (31%)~	7 (47%)~
Fat intake	4 (31%)~	7 (47%)~

^+^ Clinical data taken for the study.

*Data collected from health records.

^Data collected from questionnaire.

~Data collected from Intake 24.

### Amount of intervention received

Thirty percent (n = 8) attended all four MI sessions and 30% (n = 8) only attended one session. Nine participants only attended MI sessions during pregnancy, and three only attended postpartum sessions. The reasons for only attending postpartum sessions were: giving birth prematurely; hospitalisation; or confusion about the purpose of the study. Numbers of MI sessions attended are shown in Table 4, and the number attending each session is shown in Table 5.

**Table 4:** Number of MI sessions attended.

	Number of participants	Cumulative percentage
Intervention group	34	100%
1 or more session attended	27	79%
2 or more sessions attended	19	56%
3 or more sessions attended	14	41%
4 or more sessions attended	7	21%

**Table 5:** Number of participants attending each MI session.

	Antenatal		Postnatal	
	MI session 1	MI session 2	MI session 3	MI session 4
Number of participants who attended	24	13	17	13
Percentage of intervention group who attended	71%	38%	50%	38%

Appointments for MI sessions or data collection were declined, cancelled or missed for a variety of reasons including: did not want to or were unable to return to the hospital (*n =* 7); participant or child were unwell, injured or in hospital (*n =* 6); had forgotten (*n =* 3); out of the country (*n =* 2); or other logistical reason (*n =* 2).

Twenty-one participants in the intervention group sent messages in the WhatsApp groups. The mean number of messages sent from each participant was 15 with a median of 6 messages (range: 1 to 68). Most messages were sent between January and June 2019, which was also the time when the majority (76%, n = 16) of those participants gave birth.

#### Adaptations

During the study some adaptions were made to improve study conduct or due to logistical limitations. The main adaptation was to the randomisation process, where it became apparent that several potential participants would only participate if they were assigned their preference arm. Therefore, the study was adapted to accommodate preference to ensure the viability of the trial. Other minor adaptations were made by removing some measures (accelerometer, waist circumference and oral glucose tolerance test) due to negative participant and staff feedback or logistical constraints. Single measure diet data were collected at follow up rather than repeated, due to participant feedback. Finally, the criteria for gestational age at recruitment was broadened to address changes in hospital diagnostic processes and therefore increase recruitment.

### Primary effectiveness outcomes

Nearly half (46%, n = 6/13) of the participants in the intervention group achieved the target weight reduction of ≥5% at follow-up, compared to only 8% (n = 1/12) in the control group. The odds ratio for this was 9.4 (95% CI 0.9 to 95.9). Mean weight change between pregnancy booking and follow up at six months postpartum was -2.1kg (±9.0) in the intervention and +4.4kg (±4.9) in the control group. The mean difference between groups for weight change was -6.5kg both unadjusted (95% CI -12.6 to -0.5), indicating a difference between groups and adjusted (95%CI -12.4 to 0.2), indicating no difference between groups (see Table 6).

**Table 6:** Clinical outcomes at follow up (6 months postpartum) by on-treatment analysis using one way ANCOVA: All participants and randomised participants.

Variable		All participants			Randomised participants		
	Adjusted or unadjusted	Control (n = 12)	Intervention (n = 13)	95% confidence interval	Control (n = 11)	Intervention (n = 9)	95% confidence interval
		Mean ±SD or n (%)	Mean ±SD or n (%)		Mean ±SD or n (%)	Mean ±SD or n (%)	
**Weight**							
>5% weight loss	N/A	1 (8%)	6 (46%)	-0.69 to 0.07	1 (10.0%)	2 (25.0%)	-0.48 to 0.18
Weight change (kg)	*Unadjusted*	4.4 ±4.9	-2.1 ±9.0	-12.6 to -0.5	4.4 ±5.3	0.0 ±5.4	-9.8 to 1.0
	*Adjusted* ^6^	4.4 ±4.9	-2.1 ±9.0	-12.4 to 0.2	4.4 ±5.3	0.0 ±5.4	-11.3 to 1.2
**HbA1c**							
HbA1c (%)	*Unadjusted*	5.9 ±0.4	5.6 ±0.3	-0.5 to 0.1	5.9 ±0.4	5.7 ±0.4	-0.6 to 0.2
	*Adjusted*	5.9 ±0.4	5.6 ±0.3	-0.5 to 0.0	5.9 ±0.4	5.7 ±0.4	-0.5 to 0.1
Prediabetes^1^	*Unadjusted*	7 (58%)	4 (31%)	-0.11 to 0.65	6 (60.0%)	4 (50.0%)	-0.34 to 0.54
Diabetes^2^	*Unadjusted*	1 (8%)	0 (0%)	-0.07 to 0.23	1 (10.0%)	0 (0.0%)	-0.08 to 0.28
**Blood pressure**							
Systolic blood pressure	*Unadjusted*	125.6 ±24.9	121.0 ±20.5	-24.3 to 15.2	116.4 ±8.7	122.6 ±21.9	-10.9 to 23.2
	*Adjusted*	128.3 ±29.2	119.3 ±22.9	-31.5 to 20.2	115.5 ±10.5	125.0 ±25.9	-17.9 to 38.1
Diastolic blood pressure	*Unadjusted*	80.2 ±12.9	75.9 ±14.1	-16.0 to 7.5	75.8 ±7.3	78.0 ±13.0	-8.8 to 13.2
	*Adjusted*	81.8 ±14.8	73.9 ±15.6	-21.5 to 6.6	75.7 ±8.4	79.8 ±15.3	-14.0 to 19.6
**Diet**	* *						
Calorie intake (kcal)	*Unadjusted*	1374.5 (1152.6)	1855.7 (456.1)	-600.1 to 1562.5	1374.5 ±1152.6	1980.4 ±438.4	-701.4 to 1913.2
	*Adjusted*	965.5 (821.0)	1715.0 (578.7)	-1821.3 to 2608.6	965.5 ±821.0	1879.3 ±583.4	-2876.3 to 4019.6
Fat intake (grams)	*Unadjusted*	64.3 (49.7)	82.0 (28.6)	-34.7 to 70.2	64.3 ±49.7	79.0 ±34.0	-51.0 to 80.5
	*Adjusted*	42.5 (33.2)	89.0 (35.6)	-110.4 to 154.3	42.5 ±33.2	86.0 ±43.0	-237.6 to 218.5
**Physical activity**							
Active or moderately active		7 (58%)	7 (64%)	-0.46 to 0.34	*6 (60*.*0%)*	4 (66.7%)	-0.55 to 0.41
**Breastfeeding** ^3^							
Breastfeeding at birth		12 (100%)	11 (100%)	0.0	7 (70.0%)	4 (66.7%)	-0.44 to 0.50
Breastfeeding at 6 months		8 (67%)	8 (80%)	-0.49 to 0.23	5 (50.0%)	3 (50.0%)	-0.51 to 0.51
**Depression**	* *						
Probable depression (EPDS^4^)		5 (42%)	4 (36%)	-0.34 to 0.46	5 (50.0%)	2 (33.3%)	-0.32 to 0.66
Mild-severe depression (PHQ9^5^)		5 (42%)	4 (36%)	-0.34 to 0.46	4 (40.0%)	3 (42.9%)	-0.51 to 0.45

^1^Pre-diabetes is defined as having an HbA1c between 5.7% and 6.4%.

^2^Diabetes is defined as having an HbA1c ≥6.5%.

^3^Breastfeeding is defined as any breastfeeding (exclusive or mix feeding).

^4^EPDS = Edinburgh Postnatal Depression Scale.

^5^PHQ9 = Patient Health Questionnaire 9.

^6^Adjusted for baseline value.

When analysing the randomised only groups, 25% (n = 2) of participants in the intervention group and 10% (n = 1) in the control group achieved ≥5% weight loss at follow-up (95% CI -0.69 to 0.07). Mean weight change was 0.0kg (±5.4) in the intervention and +4.4kg (±5.3) in the control group. The mean difference between groups for weight change was -4.4kg both unadjusted (95% CI -9.8 to 1.0) and adjusted (95%CI -11.3 to 1.2). Since CIs overlapped, no differences in groups were observed.

### Secondary effectiveness outcomes

In all participants, HbA1c was 5.6% (±0.3) at follow up in the intervention group and 5.9% (±0.4) in the control group (95% CI -0.5 to 0.1 unadjusted, and -0.5 to 0 adjusted). No participants in the intervention group and 8% (n = 1/12) participants in the control group had an HbA1c ≥6.5% (commensurate with T2DM, 95% CI -0.07 to 0.23). Thirty one percent (n = 4/13) of participants in the intervention group and 58% (n = 7/12) in the control group had an HbA1c indicative of pre-diabetes (HbA1c between 5.7–6.4%; 95% CI -0.11 to 0.65).

In randomised participants, HbA1c was 5.7% (±0.4) at follow up in the intervention group and 5.9% (±0.4) in the control group (95% CI -0.6 to 0.2 unadjusted, -0.5 to 0.1 adjusted). No participants in the intervention group and 10% (n = 1/10) participants in the control group had an HbA1c ≥6.5% (95% CI -0.08 to 0.28); with 50% (n = 4/8) and 60% (n = 6/10) of participants had a HbA1c indicative of prediabetes (5.7–6.4%) in the intervention and control groups respectively (95% CI -0.34 to 0.54).

Blood pressure, daily calorie, fat intake, physical activity, breast feeding and depression were equivalent between groups in both models.

An additional observation was that those participants whose PHQ9 depression category reduced between baseline and follow up (n = 5) achieved a mean weight loss of -8.4kg ±9.2 compared to a gain of 3.0kg ±5.7 in those whose depression category did not reduce (n = 12) (95% CI 3.7 to 19.1, indicating a difference between groups).

### Effect of preference

Baseline depression scores were higher in those women who expressed a preference for the intervention. All of these women had a PHQ9 score>4, compared to 40% (n = 12) in the remaining participants. There were no other significant differences at baseline between the those who chose the intervention compared to other participants.

**Fig 3:**
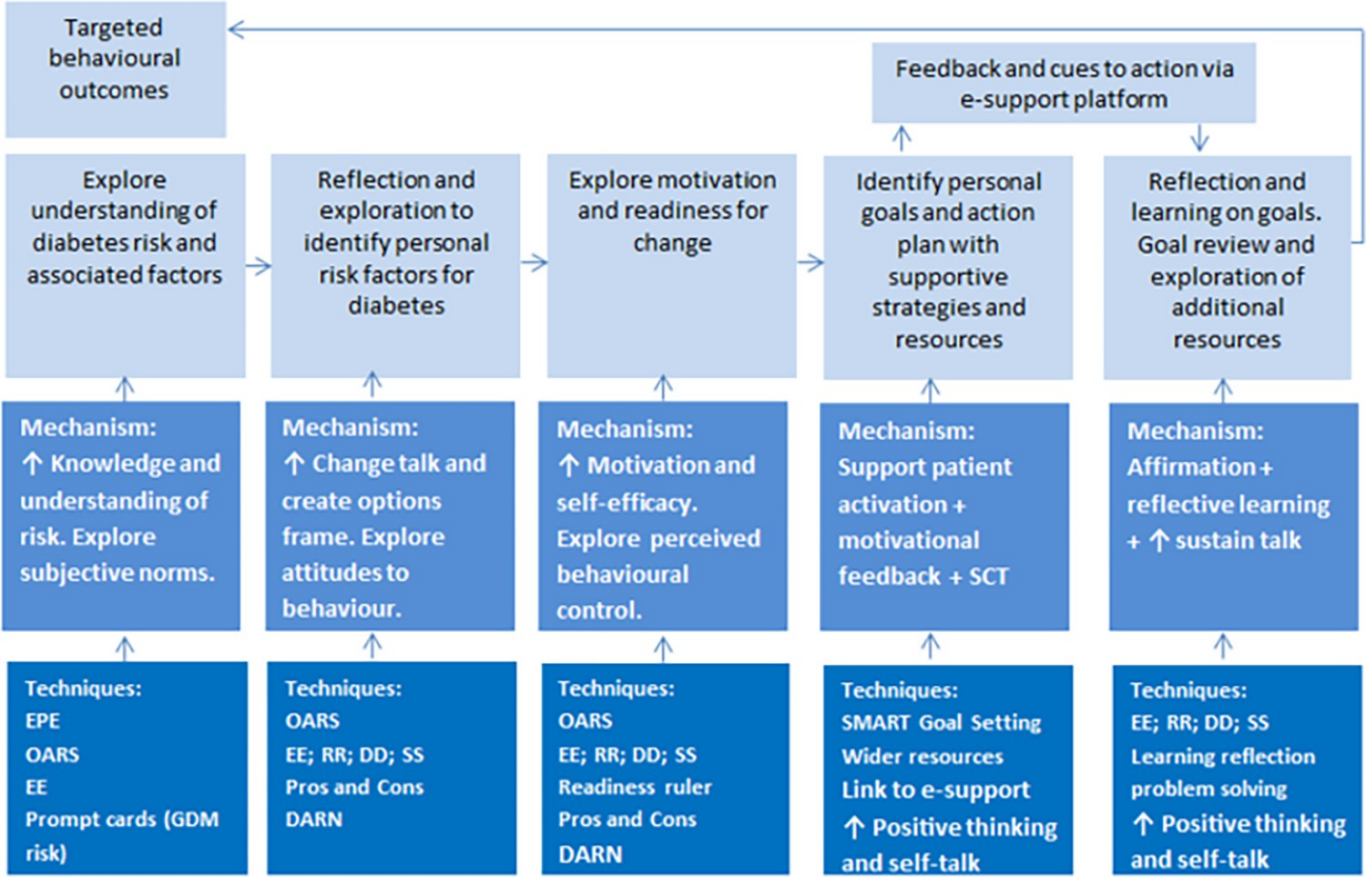
Estimated marginal means weight change between pregnancy booking and follow-up.

The analysis of preference showed the difference in proportion of participants losing ≥5% body weight between intervention and control groups was largely attributable to the PI group, which accounted for two thirds (n = 4/6) of those in the intervention group who achieved the weight loss target (see Table 1). In addition, the PI group had a mean weight change of -5.4kg, and the RI group experienced no mean change in weight (0.0kg) (see Fig 3).

## Discussion

The discussion is presented in two parts: the first considers the implications or the findings for study design and the second focuses on the outcome measures.

### Study design

Seventy two percent (n = 36) of participants consented to randomisation, which indicates feasibility for a future randomised trial. However, this would mean that over a quarter of women willing to participate in the study would be excluded. Therefore, providing women with the option to choose their allocation, while breaking the randomisation protocol thereby introducing bias, increased participation and indicates that preferences are important in determining participation in this population of women. In terms of a future trial design this introduces a dilemma. It has been suggested that preference studies can improve both external and internal validity by widening uptake and increasing adherence [21], although removing the randomisation introduces the possibility that any differences in outcome can be explained by differences between the two groups [50]. Hence, a future trial will need to decide between a RCT and a preference design. While the former will enhance internal validity, the latter could improve participation and provide understanding about the characteristics of the women who express a preference and whether this mediates the effect as suggested by our observations. If a preference design were to be considered, it may be more appropriate to offer an active control with plausible alternative to the GODDESS model. In addition, the data suggest that the intervention itself may need to be further optimised to strengthen women’s motivation for behaviour change, as these findings indicate that the intervention may only benefit those who desire it.

In relation to recruitment and retention, overall recruitment (32%) was comparable with previous similar studies recruiting during pregnancy, which have reported recruitment rates ranging from 27% to 38% [51–53], although the attrition rate was high with only 15 (44%) of the intervention group completing final data collection. Retention was much higher in the control arm (81%, n = 13). Retention rates reported in previous studies have also been higher in the control groups, ranging from 69% to 97% compared to 37% to 97% in the intervention groups [51–57]. It is also important to note the potential impact of the COVID-19 pandemic on follow-up, as three participants (all in the intervention group) did not complete the study due to COVID-19 restrictions. The other reasons provided by participants for not completing the study reflect some of the challenges faced by these women in juggling childcare and family life. The population in our study also come from a diverse inner-city area where there is a high level of geographical mobility and many have no immediate family nearby, hence women left the study either to join extended families or moved to new accommodation out of area. In a synthesis of the literature on pregnant women participating in research trials, [58] reported that pregnancy-related health issues and clinic accessibility influenced retention. These issues need to be accommodated for in both the study and intervention design of a future trial. In relation to the intervention a more flexible model of delivery may be required, potentially using virtual sessions to reduce the need for hospital appointments, accommodate childcare and other restrictions.

Another factor that seemed to be associated with recruitment and attrition was the sociodemographic backgrounds of the women. The recruitment data shows that low confidence in English language and complex questionnaires may have mediated participation. It was also noteworthy that the women who completed the study had high levels of educational attainment. This observation is in keeping with the findings of previous lifestyle intervention studies in this population [59]. However, other characteristics that have been associated with higher dropout rates in previous studies such as depressive symptoms [60], older age, women from minority ethnic groups living in deprived areas [61], and those with a higher BMI [62], were not evident in women who exited from our study prematurely. Attention needs to be given to improve recruitment of women who received less education and those with lower confidence in or knowledge of English language. This needs to include revising the way questionnaire data are collected in terms of content, format and timing. Further preparation work is also required to establish how the study and intervention can be made more attractive to these women. Consulting with women from such groups should be considered prior to a future trial as part of the PPI process.

The feasibility observations also showed some deficits in data completeness in some of the study measures. The diet data collection tool was particularly poorly completed. These data were collected through a separate online link to the Intake24 diet assessment tool embedded at the end of the questionnaire. Other than the first measure at baseline, participants were asked to complete the tool independently after their study visit. Participants’ comments on the questionnaires indicated the following reasons for poor completion: questionnaire fatigue; logistical problems with the link to the tool; the repetitive nature of the tool; and that people did not want to impart data on what they had eaten. Therefore, perhaps the Intake24 may not be an optimal measure for assessing the GODDESS intervention. Collecting self-reported data on diet and activity is recognised as a challenging issue in assessing lifestyle interventions and more invasive techniques are not feasible [63].

For a future trial, it is suggested that the measures need simplifying in terms of process and content. This may help increase data and trial completion in two ways: firstly by addressing questionnaire fatigue or difficulty in comprehension, and secondly through keeping the data collection methods in line with the motivational ethos of the intervention by personalising some of the outcomes to woman’s goals. Reducing the burden of information requested in respect of mental wellbeing, attitudes, motivation and lifestyle behaviours needs to addressed with women with GDM to ascertain their preferences and ideas for improving the data collection procedures and measures.

### Potential efficacy

The on-treatment analysis showed a potentially large benefit to intervention participants in terms of weight loss, with a mean difference of -6.5kg between groups and nearly half of the women in the intervention group achieving the target weight loss of ≥5% booking weight. In comparison, Goveia et al.’s [14] meta-analysis, which included eight studies similar to ours, reported a mean weight difference between intervention and control groups of -1.07kg (95% CI -1.43 to -0.72), in favour of the intervention group using a random effects model. The proportion of women who achieved the target weight loss in our study compares favourably to the study of Ferrara et al. [51], who reported that 38% (n = 27) in the intervention and 21% (n = 18) in the control group achieved the target weight loss. In Shyam et al.’s [64] study of a low GI diet intervention for women with previous GDM, 33% (n = 13) of the intervention group and 8% (n = 3) of the control group achieved ≥5% weight loss. The initial weight of the women in these studies was lower overall than in the GODDESS study, which might indicate larger effects in a population with higher levels of obesity. Most of the studies were larger than the GODDESS study, with less potential for bias. This is particularly relevant, as the preference participants contributed most of the effect in the GODDESS study. It is possible that those who chose the intervention group were already activated to lose weight and would have done so without the support of the intervention. Therefore, it is not clear if the larger effect size seen in GODDESS is due to higher baseline BMI and a small number of participants, or the intervention. Nevertheless, weight loss in the GODDESS study did show a potentially important clinical benefit in women who expressed an interest for the intervention.

An incidental finding of this study was the high rates of depression observed, both antenatal depression (53% (n = 20/38) with PHQ9>4) and postnatal depression (38%, n = 9/24 with PHQ9>4). The indication is that depression may be much higher in this group of women compared to the general population, where levels of antenatal depression are estimated to be around 10% [65] and postpartum depression 10–15% [66]. This observation is supported in other studies of women with GDM which have also reported relatively high levels of depression [54,67]. Depression was particularly evident in the women who had a preference for the intervention group, four of whom met the criteria for moderate to severe depression at baseline using PHQ-9. This could indicate that those with antenatal depression were more likely to want the extra support provided by the GODDESS intervention, and indeed, depression was reduced in all these women at follow-up. In addition, women whose depression appeared to reduce lost significantly more weight than women whose depression did not improve. This could indicate that an improvement in depressive symptoms mediated weight loss, or visa versa, and is something that deserves further exploration. Motivational interviewing uses counselling techniques with empathy and acceptance, and this element of the intervention may have helped mediate the mood of some participants. This effect on depression was noted in a systematic review of motivational interviewing based lifestyle interventions [68].

### Key considerations for a future trial

The study has identified a number of factors that require consideration in a future trial. These are summarised in Table 7. Key learning points indicate that a definitive trial could include a preference option with two intervention arms. The intervention arms could offer different delivery options comparing face-to-face with digital delivery and possible flexibility of intervention timing, either ante- or postnatally. Such a design could help identify different models for delivering the intervention and may boost participation. The findings indicate that data collection tools and study documents need to optimised prior to a future trial with a purposively selected PPI group in order to widen participation to include women with different levels of education and linguistic confidence. Alternative ways of providing and collecting information could include the of graphic representation of the study processes and involving a PPI representative in recruitment or data collection.

**Table 7:** Key learning points and strategies for optimising a future trial.

Key learning point	Strategies for optimising a future trial
Patient preference may impact study participation and intervention success	Include a preference option in a future trial perhaps with two intervention arms.
Women with depression may show a stronger desire to receive the intervention than other women	Consider a brief screening tool at recruitment as part of the preference design. This would help identify need together with the participant and provide the right intervention.
Depression could be a mediating factor for weight loss or visa versa	Consider this in the optimisation of the intervention and ensure depression is measured. Explore participants’ views on weight loss and depression in the evaluation.
Logistical barriers prevent women from returning for data collection and face to face intervention sessions	Consider incorporating more virtual options within both the intervention and data collection processes where possible. Further explore linking with local primary care services so that collection of clinical data can take place more locally to participants.
Women accessed the intervention at different time points across the perinatal period	Consider a cross-over preference design or allowing more flexibility on intervention time points.
Highly educated women were more likely to remain in the study and lack of confidence in English language hindered participation	Refine study processes and documentation together with purposefully selected PPI group, and consider if interpreters are an option where participants require them.
Missing or poorly completed diet data	Together with the PPI group, reconsider what we are measuring and what emotional impact this might have on participants. Simplify and reduce questionnaires.
How can the needs of those for whom the intervention was not successful or the study did not appeal be addressed?	Some of these needs may be addressed through the development of a second intervention arm, and further exploration of how these needs can be met will be conducted with the PPI group.

## Conclusions

The findings suggest that the GODDESS study was feasible and showed a favourable recruitment rate, but strategies to improve retention are needed. These could include adopting a more flexible approach to the intervention and study design. The study indicates that the intervention can support women with GDM in achieving weight loss to reduce their risk of future diabetes. This may be more effective in those women who are motivated and therefore some consideration needs to be given to how to engage women who did not lose weight in the intervention, dropped out of the study or chose not to participate.

## Supporting information

S1 Checklist CONSORT 2010 checklist of information to include when reporting a pilot or feasibility trial*. (DOC)

S1 File (DOCX)
