# Supplementary material for: The gestational diabetes future diabetes prevention study (GODDESS): A partially randomised feasibility controlled trial
Source: PLoS One. 2022 Dec 30;17(12):e0273992. doi: 10.1371/journal.pone.0273992 (PMC9803154; doi:10.1371/journal.pone.0273992)
Supplement: S1 File — (DOCX) [file pone.0273992.s002.docx]

**G**estati**o**nal **D**iabetes future **D**iab**E**te**S** prevention feasibility **S**tudy (GODDESS)

Study Protocol, Version 5

28^th^ February 2018

**Sponsor:** King’s college London (KCL) and King’s College Hospital (KCH)

**Funders:** Florence Nightingale Faculty of Nursing & Midwifery

### Study Investigators

**Chief Investigator**

**Name:** Professor Angus Forbes

**Address:** Florence Nightingale Faculty of Nursing & Midwifery, King's College London, James Clerk Maxwell Building, 57 Waterloo Road, London, SE1 8WA

**Telephone:** 020 7848 3367

**Email:** [**Angus.forbes@kcl.ac.uk**](mailto:Angus.forbes@kcl.ac.uk)

### Co-Investigator(s)

**Name:** Professor Khalida Ismail

**Address:** Department of Psychological Medicine, Weston Education Centre, King’s College London, 10 Cutcombe Road, London SE5 9RJ

**Telephone:** 020 7848 5131

**Email**: [khalida.2.ismail@kcl.ac.uk](mailto:khalida.2.ismail@kcl.ac.uk)

**Name:** Dr Katharine Hunt

**Address:** Diabetes Research Group, Weston Education Centre, King’s College London, 10 Cutcombe Road, London SE5 9RJ

**Telephone:** 020 7848 5653

**Email:** katehunt2@nhs.net

**Name:** Dr Anna Brackenridge

**Address:** St Thomas' Hospital Westminster Bridge Road London SE1 7EH

**Telephone:** 020 7188 1981

**Email:** Anna.Brackenridge@gstt.nhs.uk

**Name:** Judith Parsons

**Address:** Florence Nightingale Faculty of Nursing & Midwifery, King's College London, James Clerk Maxwell Building, 57 Waterloo Road, London, SE1 8WA

**Telephone:** 020 7848 4177

**Email:** [judith.parsons@kcl.ac.uk](mailto:judith.parsons@kcl.ac.uk)

**Name:** Helen Rogers

**Address:** Diabetes, King’s College Hospital, Ground Floor, Venetian Building, Caldecot Road, SE5 9RS

**Telephone:** 020 7848 4177

**Email:** [judith.parsons@kcl.ac.uk](mailto:judith.parsons@kcl.ac.uk)

**Sponsor**

**Name of Sponsor Organisation/s:** King's College London

**Name of Sponsor Representative:** Reza Razavi

**Address:** Room 1.8 Hodgkin Building, Guy's Campus, King's College London, London, SE1 4UL.

**Telephone:** 0207 848 6960

**Email:** [reza.razavi@kcl.ac.uk](mailto:reza.razavi@kcl.ac.uk)

**Name of Co-Sponsor Organisation/s:** King's College Hospital NHS Foundation Trust

**Name of Co-Sponsor Representative:** The R&D Office

**Address:** 161 Denmark Hill, London SE5 8EF

**Telephone:** 020 3299 1980

**Email:** [kch-tr.research@nhs.net](mailto:kch-tr.research@nhs.net)

**1. Introduction**

1.1 Background

1.2 Prevention of T2DM in high-risk groups through lifestyle modification

1.3 Prevention of T2DM after GDM

1.4 Why this group?

1.5 Current diabetes prevention interventions

1.6 Qualitative research

**2 Trial objectives, design and statistics**

2.1. Trial objectives

2.2 Intervention

2.3 Trial design and flowchart

2.4 Outcome measures

2.5 Trial statistics and analysis

**3. Sample size, selection and withdrawal of subjects**

3.1 Inclusion criteria

3.2 Exclusion criteria

3.3 Criteria for Premature Withdrawal

**4. Study procedures**

4.1 Informed consent procedures

4.2 Screening Procedures

4.3 Randomisation Procedures

4.4 Schedule of Treatment for each visit

4.5 Follow up procedures

4.6 End of Study Definition

**5. Laboratories**

5.1 Central/local Laboratories

5.2 Sample collection/labelling logging

5.3 data Recording/Reporting

**6. Assessment of Safety**

6.1 Ethics Reporting

**7. Trial Steering Committee**

**8. Ethics & Regulatory Approvals**

**9. Data Handling**

9.1 Confidentiality

9.2 Case Report From

9.3 Record retention and Archiving

9.4 Compliance

9.5 Clinical Governance issues

9.6 Non Compliance

**10. Finance & Publication Policy**

### Study Synopsis

| **Title** | **G**estati**o**nal **D**iabetes future **D**iab**E**te**S** prevention **S**tudy – feasibility trial |
| --- | --- |
| **Protocol Short Title/Acronym** | GODDESS |
| **Protocol Version number and Date** | Version-4  Date: 17/01/18 |
| **Study Phase if not mentioned in title** | N/A |
| **Is the study a Pilot?** | No |
| **Study Hypothesis** | The primary hypothesis (H^O^1) for the study is that a lifestyle intervention to increase physical activity and promote weight loss in women with recent gestational diabetes mellitus (GDM) is feasible |
| **Study Duration** | 18 months |
| **Methodology** | Feasibility randomised controlled trial (RCT) |
| **Sponsor name** | Mr. Reza Razavi |
| **Chief Investigator** | Professor Angus Forbes |
| **REC number** | **177238** |
| **Medical condition or disease under investigation** | Risk of type two diabetes in women with previous gestational diabetes |
| **Purpose of clinical trial** | To test the feasibility of a trial to improve diet, increase physical activity and promote weight loss in women with recent GDM, with a view to preventing or delaying T2DM |
| **Primary objective** | To explore the acceptability of a lifestyle intervention to increase physical activity and promote weight loss in women with recent GDM, and test the feasibility of the trial |
| **Secondary objective (s)** | To assess preliminary efficacy of the intervention |
| **Number of Subjects/Patients** | 60 |
| **Trial Design** | Feasibility two-armed parallel group RCT |
| **Endpoints** | Once the primary outcome variables have been appropriately measured and data analysis completed, this marks the end of the study. |
| **Main Inclusion Criteria** | - aged ≥18 years - diagnosed with GDM (NICE cirteria) - ≤30 weeks pregnant - able to speak and understand English - body mass index (BMI) of ≥ 25kg/m^2^ (or ≥22 kg/m^2^ if Asian) |
| **Statistical Methodology and Analysis** | Feasibility will be assessed through the process evaluation.  For the preliminary efficacy outcomes, between group and group*time interactions will be used to estimate the intervention effects (analysis of variance). The analysis will be performed using SPSS by intention to treat. Further regression analysis will be used to adjust for any potential confounding factors not factored into the stratification. |

# Glossary of Terms and Abbreviations

BCT Behaviour change technique

CI Chief Investigator

CRF Case Report Form

GDM Gestational diabetes mellitus

MREC Main Research Ethics Committee

NHS R&D National Health Service Research & Development

PI Principle Investigator

QA Quality Assurance

QC Quality Control

Participant An individual who takes part in a clinical trial

RCT Randomised Controlled Trial

REC Research Ethics Committee

SAE Serious Adverse Event

SDV Source Document Verification

SOP Standard Operating Procedure

SSA Site Specific Assessment

TMG Trial Management Group

TSC Trial Steering Committee

T2DM Type 2 Diabetes Mellitus

### 1. Introduction

- 1. **Background**

GDM is a common condition, occurring in around 5% of pregnancies [1], and is increasing [2]. Women with GDM are over seven times more likely than women with normoglycaemic pregnancies to develop T2DM [3], and their risk is estimated at 30% within 5-16 years of pregnancy [4]. Compared to women with normoglycaemic pregnancies, women with GDM have a 30-84% increased chance of another episode of GDM [5], and children of women with diabetes in pregnancy are more likely to become obese or develop T2DM [6]. GDM is associated with adverse fetal, infant and maternal pregnancy outcomes [7], and T2DM is associated with increased morbidity and mortality [8].

- 1. **Prevention of T2DM in high-risk groups through lifestyle modification**

Large-scale studies have shown that T2DM can be prevented or delayed in high-risk populations through lifestyle modifications after psychological or educational interventions. Findings from the Diabetes Prevention Program (DPP) Research Group show that a lifestyle intervention reduced the incidence of diabetes in a high risk group by 58% [9]. Tuomilehto et al. [10] also found a 58% reduction in diabetes incidence after individualised lifestyle counselling in the intervention group compared to controls.

- 1. **Prevention of T2DM after GDM**

A sub-analysis of the DPP data showed that women with GDM (mean 12 years prior to the study) experienced a 50% reduction in incidence of T2DM after the lifestyle intervention [11]. However, other than this, little research has been conducted on prevention of T2DM specifically after GDM. There are a small number of pilot randomised controlled trials (RCTs) exploring the impact of lifestyle interventions on behavioural and metabolic markers. These studies have shown either no improvements [12]or some non-significant improvements [13-15], with one study finding a significant decrease in dietary fat intake in the intervention group [14]. These are largely pilot studies, none of which were UK based and the interventions were not modelled in a GDM population prior to implementation.

- 1. **Why this group?**

We will target women with recent GDM, as the cumulative incidence of T2DM after GDM shows the largest increase in the 5 years following pregnancy [16]. In addition, women are more likely to have another pregnancy in this period rather than later, meaning an intervention could help prevent a further episode of GDM. We will recruit women during pregnancy, when they are in frequent contact with health services and are already activated in relation to their health behaviours. Women can be receptive to change in the period around childbirth, and an intervention at this time could offer additional information and support to address the child’s diabetes risk by encouraging healthy behaviours for the whole family.

- 1. **Current diabetes prevention interventions**

The current gold-standard model for diabetes prevention based on the interventions used in large national programmes, is detailed in the IMAGE guideline (Development and *Im*plementation of *a* European *G*uidelin*e* and Training Standards for Diabetes Prevention) [17] . While this guidline provides a model for lifestyle intervention including a supportive programme of behaviour change, our preliminary work indicates this approach needs to be adapted for women with previous GDM. Previous studies with women with GDM have demonstrated increased barriers to engaging in lifestyle interventions or healthy behaviours postpartum, including the emotional stress of adjusting to motherhood, lack of time and childcare responsibilities [18, 19]. Pilot studies exploring the impact of lifestyle interventions post GDM [12, 15] cited recruitment and retention as challenges, and another [13] found that women felt the intervention, which ran from 6 weeks to 18 weeks postpartum, was too close to their baby’s birth. It is, therefore, important to adapt existing interventions to address the context of motherhood, and carefully consider the timing and delivery of the intervention to fit with the woman’s priorities. In addition, many of the general prevention intervention studies targeted older groups of people with impaired glucose regulation and are not congruent with the health perceptions and lifestyles to women with young infants. None of the pilot studies exploring lifestyle interventions for women with GDM were UK-based, and it is important to identify an intervention that is acceptable to the population group and can be easily translated into the NHS in a cost-effective manner.

- 1. **Qualitative research**

We have carried out a meta-synthesis of current qualitative studies of women with GDM [20] as well as conducting our own qualitative work with interviews and focus groups involving 50 women with previous GDM. The data indicates that women are keen to receive individualised lifestyle support after the birth, and from their accounts we have elicited a number of important facilitators and barriers to their adoption of positive health behaviours. We have used these data to adapt our intervention to enhance its sensitivity to the target population.

**2. Trial objectives, design and statistics**

### 2.1. Trial Objectives

The aim of the study is to explore acceptability of a lifestyle intervention to improve diet, increase physical activity and promote weight loss in women with recent GDM with a view to preventing or delaying T2DM, and assess the feasibility of the trial prior to a larger clinical trial.

**2.2 Intervention**

GODDESS aims to prevent T2DM and further episodes of GDM by increasing healthy eating, physical activity and weight loss. It has been adapted from the IMAGE (Development and **IM**plementation of **A** European **G**uid**E**line and Training Standards for Diabetes Prevention) diabetes prevention toolkit, which is based on a synthesis of current evidence for diabetes prevention (Lindström et al., 2010)- <http://www.image-project.eu/pdf/final_version_of_toolkit-perfect.pdf>. The IMAGE toolkit includes intervention frameworks for screening and for population, community and individual level intervention. The GODDESS intervention focuses on individual level intervention. IMAGE utilises a theoretically grounded behaviour change model that aims to prevent T2DM by:

- improving dietary intake (by reducing fat and refined carbohydrate intake, and increasing fibre intake);
- increasing physical activity (to over 30 minutes of moderate activity per day);
- promoting weight loss (≥ 5% loss).

GODDESS has adapted this model to reflect the needs of women with GDM based on our research into their needs. These include: attending to the psychological response of a GDM pregnancy; tailoring interventions to reflect the stage in pregnancy and the developmental stage of the infant (e.g. breast feeding, infant weaning and being able to exercise with their infant); integrating diet and physical activity within the context of the infant, child and wider family life; contextualising women’s diabetes and health risks in relation to infant and family well-being; and considering the particular challenges that come with motherhood.

We have also adapted the activity and weight loss targets to reflect NICE guidance on weight loss and pregnancy (<https://www.nice.org.uk/guidance/ph27/chapter/1-recommendations#recommendation-3-supporting-women-after-childbirth>.). Our targets for physical activity during pregnancy have been modified to concord with the NICE guideline which states that: women who have not exercised routinely should begin with no more than 15 minutes of continuous exercise, three times per week, increasing gradually to daily 30-minute sessions. We have also amended the weight reduction target to state that weight loss should only be attempted at 6-8 weeks postpartum with the aim of ≥ 5% reduction by 12 months from that point. The pregnancy activity and dietary targets have also been informed by the findings of the UPBEAT study [21]. This study was undertaken by members of our team and targeted obese women in pregnancy and reported reduced gestational weight gain (GWG) and increased physical activity without any adverse events [22]. The intervention has been further adapted and designed following a structured format to facilitate adoption within current NHS care delivery systems. The target behavioral outcomes for each phase of the intervention are identified in table 1. These are the global targets, each woman will set their own individual goals.

| Table 1. Behavioural outcomes | | | |
| --- | --- | --- | --- |
| *Intervention Phase* | *Target behavioral outcomes* | | |
|  | *Physical Activity* | *Diet* | *Weight loss* |
| Pregnancy | 1. 15 minutes of moderate physical activity increasing to 30 minutes (brisk walking, swimming) 2. Walking increase by 20% aiming for 10,000 steps per day | 1. Increase: fruit and veg intake; whole grain foods. 2. Reduce snacks and sugary drinks, refined carbohydrates and portion sizes. 3. Eat breakfast. 4. No calorie targets will be set for pregnancy | Weight loss not applicable but relative GWG to be managed as per baseline BMI:  Baseline BMI 25-29, GWG ≤ 9kg; BMI ≥30, GWG ≤ 6kg*. |
| Early post-partum (0-3 months) | 1. 30 Minutes of moderate physical activity 3 times per week increasing to daily. 2. Walking 10,000 steps per day | 1-3 as per pregnancy +   1. Breast feeding 2. Daily calorie target = 1,200 (+330 if breast feeding). | 1-2KG weight loss per week from 2 months post-partum. |
| Up to first year of life | 1-2 as per early post-partum | 1-3 as per pregnancy +   1. Daily calorie target = 1,200 (+400 if breast feeding). | ≥5% weight loss from 2 months post-partum. |
| *= Cedergren, M. (2007) Optimal gestational weight gain for body mass index categories. Obstet Gynecol. 2007 Oct;110(4):759-64. | | | |

The intervention is comprised of 5 face to face interactions (60 minutes) across the antenatal and postpartum periods delivered using motivational interviewing (MI) techniques. The focus of the sessions are targeted to reflect the changing context of needs during pregnancy and the postpartum period. The intervention is supported by a facilitated ‘WhatsApp’ group, motivational text messages, a pedometer and a website. An overview of the GODDESS intervention is provided in figure 1.

***Figure 1. Overview of GODDESS intervention***

***
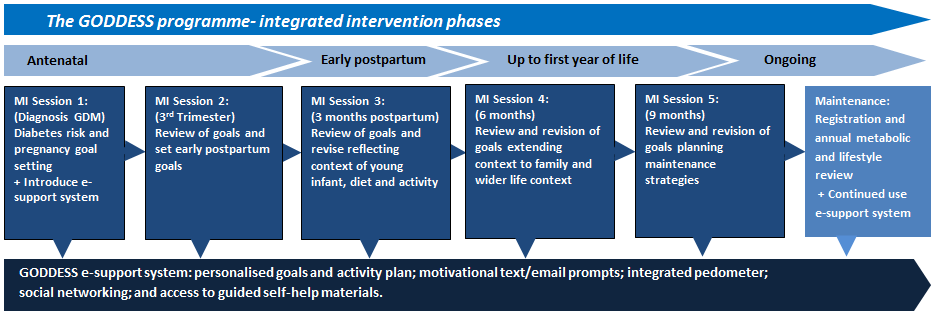
***

***2. Theoretical framework and behaviour change techniques.***

GODDESS is underpinned by the following theoretical models for behavior change described in the IMAGE prevention toolkit. These include: the theory of planned behaviour and social cognitive theory. These models are integrated into a ‘self-regulatory’ framework incorporating: motivation; specific goal-setting; an action plan; feedback on performance; and goal review (Greaves et al, 2011)- see figure 2.

***Figure 2. Behaviour change model***


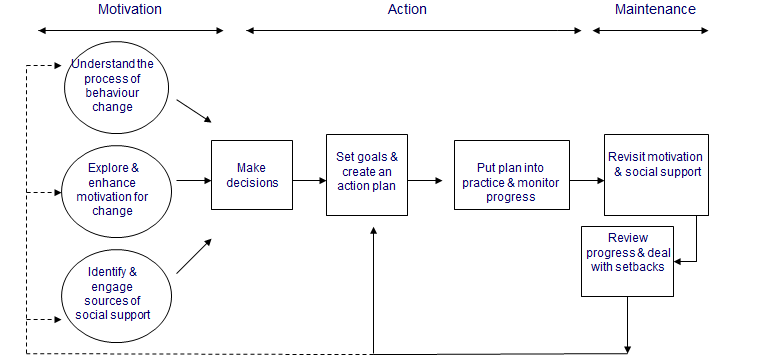


GODDESS also incorporates the 12 behaviour change techniques (BCT) identified Abraham & Michie’s (2008) BCT taxonomy:

- Provide information on consequences
- Prompt intention formation
- Prompt barrier identification
- Prompt specific goal setting
- Prompt review of behavioural goals
- Prompt self-monitoring of behaviour
- Teach to use prompts or cues
- Agree on behavioural contract
- Use follow-up prompts
- Plan social support or social change
- Relapse prevention

The principal technique used to deliver the theoretical model and BCT for GODDESS is motivational interviewing (MI). MI is a method that works on facilitating and engaging intrinsic motivation within the person within a co-productive (intervention provider and recipient) relationship in order to change behavior. MI is a goal-oriented, client-centered counseling style for eliciting behavior change by helping the person to explore and resolve ambivalence (Miller and Rose, 2009). MI is a proven approach for achieving weight loss in those who are overweight or obese (Armstrong et al, 2011). We are using MI to enhance the engagement of the women in making lifestyle change by: increasing their desire for change; addressing and reducing resistance to change; developing their confidence (self-efficacy) and commitment to change; setting realistic SMART goals with an individual action plan; reflecting and learning on goals; and by eliciting and mobilising social support and other resources to help them achieve and sustain change. All the face-to-face sessions will be conducted following an MI approach. These sessions will supplemented by the GODDESS e-support system which has also been designed following the same principles for behavior change with motivational feedback on performance, prompts and cues to action (see appendix 1). The active psychological mechanisms/techniques and their relationship to the overall behavior change model for GODDESS are detailed in Figure 2.


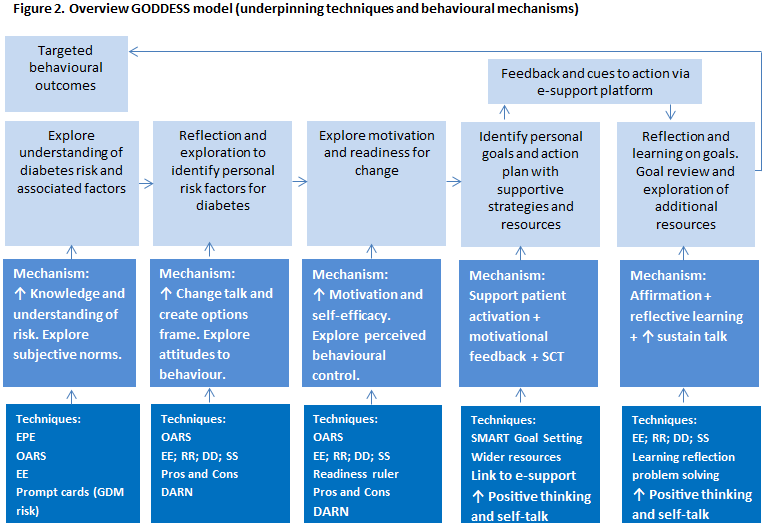


| **Key for techniques** | |  |
| --- | --- | --- |
| **EPE** | **‘Elicit, Provide, Elicit’** | |
| **OARS** | **‘Open questions, Affirmations, Reflections, Summaries’** | |
| **EE** | **‘Express Empathy’** | |
| **RR** | **‘Roll Resistance’** | |
| **DD** | **’Develop Discrepancy’** | |
| **SS** | **‘Support Self-efficacy’** | |
| **SMART** | **Simple, Measurable, Achievable, Relevant, Time based** | |
| **SCT** | **Social Control Theory** | |

The GODDESS intervention also incorporates the following specific lifestyle enhancement approaches: the frequency intensity time type (FITT) activity framework; specific dietary principles for short term weight loss and for maintenance; ‘eat clever’ principles; and recording and monitoring activities (Greaves et al, 2011). To enhance the physical activity element of the intervention the women will be given a pedometer with individualised step targets. The pedometer is delivered by a wearable technology that communicates with the e-support system to allow the women to see their progress and to trigger motivational feedback. Again there is evidence from meta-analyses of clinical trials showing that pedometers can increase activity and enhance weight loss, their effect is greatest when embed in a broader intervention of intervention such as GODDESS (Richardson et al, 2007; Bravata et al, 2007). Prompt and option cards with simple visual images are used to trigger discussion and help women consider their individual goals.

Consideration has also been given to socio-cultural adaptability of the intervention as it will be implemented in a range of different populations. The key strategies for this are to target transferable behavior patterns related to common lifestyle behaviours of relevance to all populations and to draw on the expertise of the educators to consider local and individual level factors related to the women’s socio-cultural contexts. The core principle is to keep behavioral goals simple and relevant and to support the women to identify strategies that work for them. In terms of behavioural goals the focus is on: the consumption of high energy drinks, reducing between meal snacks; reducing portion sizes; and increasing physical activity in their everyday routines. The educators will also be able to help women identify local resources (such as fitness programmes) that are appropriate to their cultural references.

### 2.3 Trial Design & Flowchart

The study has been designed as a feasibility two-armed parallel group RCT with integrated process evaluation, as shown in the adapted CONSORT diagram in Figure 1. An RCT has been chosen because: (1) this is the gold standard design to determine causal associations, (2) a control group design is necessary because changes related to pregnancy and the postpartum period might impact outcomes (e.g. weight change) and make between group interactions less reliable than group*time interactions, and (3) the study will provide preliminary information on feasibility with regards to recruitment and retention, acceptability and outcome measures, before undertaking a large-scale RCT.

**Figure 1. CONSORT flowchart**

**29-32 weeks' gestation:** Dietician or researcher provide information to potentially eligible participant during group education session. Or clinician providing routine care to approach potentially eligible participants at first clinic appointment - brief verbal and written information given and verbal permission for researcher to contact sought

**Baseline / randomisation visit (29-33 weeks' gestation):** Eligibility confirmed; written informed consent (if not previously obtained); randomisation; demography; questionnaires; anthropometry; blood pressure; blood sample; OGTT, provided with 7 day accelerometer and instructions

**DECLINED or INELIGIBLE**

Routine demographic data recorded if agreed

**29-33 weeks' gestation:** Eligibility screening, responding to questions about the study and taking informed consent (researcher or clinician conducting routine appointment)

**Face-face MI session 5 (9 months postpartum)**

**Follow up data visit (9 months postpartum):** Questionnaires; anthropometry; blood pressure; blood sample; OGTT; provided with 7 day accelerometer

**Face-face MI session 4 (6 months postpartum)**

**Face-face MI session 3 (3 months postpartum)**

**DROP OUT**

Pre-collected data used if agreed

**Interim data visit (3 months postpartum):** Questionnaires; anthropometry; blood pressure; blood sample; OGTT; provided with 7 day accelerometer

**Face-face MI session 2 (33-37 weeks’ gestation)**

**CONTROL GROUP:**

Routine care

**INTERVENTION GROUP**

**Face-face MI session 1 (29-33 weeks’ gestation)**

**Given access to WhatsApp group, pedometer and website**

|  | Recruitment | Screening | Face-to-face session 1 | Face-to-face session 2 | Face-to-face session 3 | Face-to-face session 4 | Face-to-face session 5 | Interview |
| --- | --- | --- | --- | --- | --- | --- | --- | --- |
| Patient information | X |  |  |  |  |  |  |  |
| Inclusion and exclusion check | X |  |  |  |  |  |  |  |
| Patients who meet the study inclusion criteria | | | | | | | | |
| Informed consent |  | X |  |  |  |  |  |  |
| Demographics |  | X |  |  |  |  |  |  |
| Blood sample |  |  | X |  | X |  | X |  |
| Weight, height, waist circumference |  |  | X |  | X |  | X |  |
| Depression (PHQ 9) |  |  | X |  | X |  | X |  |
| Edinburgh Postnatal Depression Scale |  |  | X |  | X |  | X |  |
| Quality of life (EuroQol EQ-5D-5L) |  |  | X |  | X |  | X |  |
| Body Appreciation Scale |  |  | X |  | X |  | X |  |
| Three Factor Eating Questionnaire |  |  | X |  | X |  | X |  |
| Dietary Change Motivation Scale |  |  | X |  | X |  | X |  |
| **Risk Perception Survey for Developing Diabetes** |  |  | X |  | X |  | X |  |
| Multiple pass 24 hour diet recall (online) |  |  | X |  | X |  | X |  |
| Adapted GPAQ physical activity questionnaire |  |  | X |  | X |  | X |  |
| Sleep |  |  | X |  | X |  | X |  |
| Infant feeding intentions scale |  |  | X |  |  |  |  |  |
| Infant feeding |  |  |  |  | X |  | X |  |
| Infant data |  |  |  |  | X |  | X |  |
| Data from motion watch |  |  | X |  | X |  | X |  |
| What’sApp group and fitbit usage |  |  |  |  | X |  | X |  |
| Process evaluation |  |  |  |  |  |  |  | X |

**X = all participants**

**X = only participants randomised to intervention group**

**2.4 Outcome Measures**

The primary outcome is feasibility of the trial. This will be assessed during the process evaluation through:

- recruitment rate
- attrition rate
- reasons for non-acceptance
- trial procedures for randomisation
- amount of staff time required
- fidelity, measured through a self-report check-list
- staff feedback on feasibility
- participants’ satisfaction with the intervention
- acceptability of research procedures, data collection and overall experience of the trial

The secondary outcomes will assess preliminary efficacy of the intervention. We will measure the following :

- Oral Glucose Tolerance Test (OGTT) to assess differences ≥1 mmol/l in either fasting or two hour postprandial glucose
- Incident T2DM and a subsequent GDM pregnancy (NICE criteria)
- Relative and absolute weight loss at multiple time points considering the number of women achieving significant weight loss (>5%) at 3 and 9 months postpartum from a baseline of the booking appointment weight
- Lifestyle factors, following the Medical Research Council (2012) diet and physical activity measurement toolkit and from accelerometer
- Other metabolic and anthropometric factors will include HbA1c, waist circumference and blood pressure
- Psychological factors; depression/anxiety (PHQ9) and postnatal depression (Edinburgh postnatal depression scale)
- Health related quality of life- EuroQol EQ-5D-5L will be used to assess health related quality of life
- Diabetes risk perception (Risk Perception Survey for Developing Diabetes used in the DPP [9]).
- Infant factors- infant feeding intentions, breast feeding duration, weight gain and growth, and measure of attachment.
- Attitudes to body [23], eating behaviour [24] and motivation for dietary change [25].
- Additional data will include: age, ethnicity, deprivation, employment, education, pre-pregnancy BMI, parity, previous GDM and birth outcome data

### 2.4 Trial Statistics and Analysis

We do not yet have the information necessary for a formal power calculation. The aim of this study is to measure feasibility including estimating the effect size and standard deviation to formulate a power calculation for a definitive trial. The sample size of 30 in each arm would be able to detect an effect size of 0.8 in a continuous variable such as weight loss, and if allowing for a 20% attrition rate (n=16), 0.9. Therefore we do not expect do detect differences unless the effect size is large.

For the preliminary efficacy outcomes, between group and group*time interactions will be used to estimate the intervention effects (analysis of variance). The analysis will be performed using SPSS by intention to treat. Further regression analysis will be used to adjust for any potential confounding factors not factored into the stratification.

### 3. Sample Size, Selection and Withdrawal of Subjects

A convenience sample of 60 women with current GDM, 30 in each arm will be recruited. King’s College Hospital antenatal diabetes clinic sees around 200 new patients with GDM a year, equating to 17 a month, and Guy’s and St Thomas’ Hospitals see 700 (58 a month). Participants will be recruited opportunistically at their routine clinic appointment upon diagnosis of GDM, group education session the following week, or at a following clinic visit, by a diabetes nurse, consultant or dietician. We estimate that with a 30% recruitment rate we can recruit our sample within 3 months, or with a 15% recruitment rate we can recruit within 6 months. The intervention will last approximately 12 months (depending on gestational stage at diagnosis – usually this is around 28 weeks but can be earlier), with follow-up stopping at 9 months postpartum. We aim to start recruitment, ethical approval permitting, in March 2018, and finish data collection in September 2019. We expect the data to be analysed by December 2019.

Pregnant women have been chosen because: (1) previous research and our qualitative research indicates this is the best time to recruit women to start providing support, (2) women are already engaged with health services so may be easier to recruit, (3) women may be more receptive to behaviour change support during pregnancy, and (4) the highest cumulative incidence of T2DM after GDM is in the first 5 years postpartum, so any intervention should start as soon as possible after identification of GDM.

Potential participants will be identified by the clinical team working at KCH and GSTT. Potential participants will be given the participant information sheet explaining the purpose of the study. Participants who indicate a potential interest in the study will be given the opportunity to consider their participation in the study before being scanned by the researcher for full eligibility using the eligibility screening form. After screening, if the patient is eligible they will undergo consenting, either on the same day or another day at their convenience. Participants may withdraw their interest in the study at any point during the screening and consenting process and their details will not be stored.

**3.1 Inclusion Criteria**

- aged ≥18 years
- Diagnosed with GDM (NICE criteria)
- ≤30 weeks pregnant
- able to speak and understand English
- Access to the internet
- body mass index (BMI) of ≥ 25kg/m^2^ (or ≥22 kg/m^2^ if Asian)

**3.2 Exclusion Criteria**

- Unable to consent
- Under 18 years
- BMI <24, or <22 in Asian ethnic groups
- ≥31 weeks pregnant
- Unable to speak and understand English
- Serious mental illness
- No access to the internet

**3.3 Criteria for Premature Withdrawal**

Participants will be free to withdraw from the study at any time without giving reasons and without prejudicing further treatment. If the participant no longer meets the inclusion criteria, for example they are diagnosed with type1 or type 2 diabetes, or they become pregnant they will be withdrawn from the study. Similarly, if the participant is unwell and unable to comply with the other aspects of the protocol or if a participant were no longer able to give informed consent during the study, they would be withdrawn. In all cases where possible and with permission from the participant, any data already collected up until the time of withdrawal would be kept and will be used in the data analysis unless the participant requests otherwise. This will clearly be outlined in the Participant Information Sheet and itemised in the Patient Consent Form.

### 4. Study procedures

**4.1 Informed Consent Procedures**

Potentially eligible participants attending out-patients diabetes clinics at KCH and GSTT will be given the Patient Information Sheet by the clinician. They will then be given at least 48 hours to consider their participation in the study and discuss the decision with family, friends and their care team. A member of the research team will then contact them by telephone to answer any additional questions, verify their understanding of what is involved and confirm their interest in study participation and to arrange for the screening appointment. If the participant is interested in taking part in the study the researcher will gain written consent and recruit to the study.

For each consent form participants will write their initial next to each statement of the Patient Consent Form and sign next to their name. A suitably trained and experienced member of the research team who is authorised on the Delegation Log will then sign the self-duplicating Patient Consent Form, retain two parts (to be stored securely by the researchers and in the hospital medical notes), and give back one part to be retained by the participant. The right of the participant to refuse to participate without giving reasons will be respected. Recruited participants will be consented prior to any data collection.

All participants are free to withdraw at any time from the study without giving reasons and without prejudicing further treatment. No further data will be collected. Data already collected will still be used in the data analysis unless the participant requests otherwise; this will clearly be outlined in the Participant Information Sheet and itemised in the Patient Consent Form.

**4.2 Screening Procedures**

*Screening Visit* (20-30 minutes)

Potential participants will screened for eligibility using the eligibility checklist. They will then be offered the opportunity to ask questions about the study. If they are eligible and happy to proceed, written consent will be taken.

*Data collection visit 1*

The following baseline data will be collected:

- Blood sample (HbA1c) (OGTT data will be taken from patient record)
- Blood pressure
- Weight, height, waist circumference (BMI at booking will be taken from patient record)
- Demographics
- Quality of life (EuroQol EQ-5D-5L)
- Depression (PHQ9)
- Edinburgh Postnatal Depression Scale
- Body Appreciation Scale
- Dietary Change Motivation Scale
- Three Factor Eating Questionnaire
- **Risk Perception Survey for Developing Diabetes**
- Multiple pass 24 hour diet recall (online)
- Adapted GPAQ physical activity questionnaire
- Sleep questionnaire
- Infant feeding intentions scale
- Accelerometer

*Data collection visit 2* (3 months postpartum)

The following interim data will be collected:

- Blood sample (HbA1c)
- OGTT
- Blood pressure
- Weight, height, waist circumference (BMI at booking will be taken from patient record)
- Demographics
- Quality of life (EuroQol EQ-5D-5L)
- Depression (PHQ9)
- Edinburgh Postnatal Depression Scale
- Body Appreciation Scale
- Dietary Change Motivation Scale
- Three Factor Eating Questionnaire
- **Risk Perception Survey for Developing Diabetes**
- Multiple pass 24 hour diet recall (online)
- Adapted GPAQ physical activity questionnaire
- Sleep questionnaire
- Infant feeding
- Infant data (taken from patient records)
- Accelerometer

*Data collection visit 3* (9 months postpartum)

The following follow-up data will be collected:

- Blood sample (HbA1c)
- OGTT
- Blood pressure
- Weight, height, waist circumference (BMI at booking will be taken from patient record)
- Demographics
- Quality of life (EuroQol EQ-5D-5L)
- Depression (PHQ9)
- Edinburgh Postnatal Depression Scale
- Body Appreciation Scale
- Dietary Change Motivation Scale
- Three Factor Eating Questionnaire
- **Risk Perception Survey for Developing Diabetes**
- Multiple pass 24 hour diet recall (online)
- Adapted GPAQ physical activity questionnaire
- Sleep questionnaire
- Infant feeding
- Infant data (taken from patient records)
- Accelerometer

Interview: 9 months postpartum (1 hour)

A structured interview to explore the following will be conducted by a researcher and take place within four weeks of session 5:

- Reasons for drop-out (if applicable)
- Feedback on and satisfaction with the MI intervention
- Feedback on use of the WhatsApp group and any apps used
- Feedback on using the pedometer
- Acceptability of research procedures, data collection and overall experience of the trial

**4.3 Randomisation Procedures**

Participants will be randomised using a computer-generated random number table in blocks of 10. Randomisation will be supported by the Clinical Trials Unit. Double blinding is not possible because the participants will know if they receive an intervention or not. However, this potential source of bias will be considered in the analysis and would be eliminated in a definitive trial. In order to address attrition bias, participants in the control group will be provided with diabetes and health information at follow-up (after completion of measurements).

**4.4 Schedule of Treatment for each visit**

Data from consenting patients who meet the inclusion criteria and are randomised to the intervention group will receive the following treatment.

| **Table 1: Face-to-face motivational interviewing (MI) Sessions** |
| --- |
| **Session 1: Diagnosis - 2 weeks post diagnosis of GDM (60 mins)** |
| Focus:   - Allow patient to ventilate and express their thoughts and feelings about the diagnosis and their concerns and health beliefs. - Elicit patient’s understanding of diabetes risk and provide simple explanation- confirm understanding. - Elicit what the patient thinks will reduce their risk and provide menu of potential actions that may reduce risk- explore pros and cons with patient. - Encourage patient to choose areas of action, focus on discrete behaviours such as: consumption of sugary drinks, snacks, fast-food, portion sizes and increasing walking. - Explore importance and confidence of action areas- develop self-efficacy as required. - Set pregnancy lifestyle goals (diet and physical activity) with patient using SMART model. - Get patient to identify what personal and social resources they will use in achieving goals. - Introduce and fit patient with the wearable technology (pedometer). - Introduce participant to WhatsApp group   Materials:   - Prompt-card detailing factors contributing to their risk of diabetes. - Prompt-card benefits to their health in pregnancy and for their baby. - Prompt-card with menu of lifestyle steps they can take in pregnancy. - Credit-card sized summary with key messages and web address for additional support. - Pros and cons of breast feeding in terms of diabetes risk for patient and baby.   Targets:   - Patient understands diabetes risk and contributing factors. - Patient motivation and activation. - Patient identifies diet and activity goals. - Patient is familiar with can use pedometer. - Patient is familiar with and can access WhatsApp group. |
| **Session 2:Third trimester (60 minutes)** |
| Focus:   - Reflect on goals from Session 1 affirming what they have achieved and learning reflectively from their experiences, attending to self-efficacy defects and directing to ongoing goals. - Elicit health beliefs, fears and worries, what is going well and what has gone less well. - Elicit what the patient thinks will be important in reducing their risk after pregnancy. - Share ideas on prevention activities in first 3 months postpartum using prompt cards- reflect on infant and family health context. - Review pedometer use. - Set new SMART goals with patient on prevention activities for first 3 months postpartum. - Review use of WhatsApp group. - Record new patient goals on intervention database and update personal plan. - Explain follow-up support to be provided.   Materials:   - Prompt-card with menu of lifestyle areas for early postpartum, including healthy eating tips, breast feeding and strategies for being active with a young infant.   Targets:   - Patient remains motivated and engaged. - Patient identifies 0-3 month postpartum diet and activity goals. - Patient is using pedometer and WhatsApp group. |
| NB. Pregnancy outcomes will be reviewed prior to progression to session 3. |
| **Session 3: 3 months postpartum (60 minutes)** |
| Content:   - Reflect on birth experience and on early postpartum goals, affirming what they have achieved and learning reflectively from their experiences, attending to self-efficacy defects and directing to ongoing goals. - Elicit what the patient thinks will be important going forward to sustain and adapt their lifestyle as the infant grows and their needs change- e.g. weaning and changing socialisation. - Elicit what social and wider resources can help them. - Elicit ways in which the wider family might develop an enhanced lifestyle. - Share ideas on prevention activities from the 3-6 month period using prompt cards- reflect on infant and family health context. - Set SMART goals with patient on prevention activities for first 3-6 month postpartum period. Goals can either be specific lifestyle goals or if patient is not yet ready for action they can be reflective exercises designed to enhance motivation. - Review goal attainment data and pedometer use and explore with patient strategies to increase steps- set a new target. - Discuss patients weight with them in a positive way and any relevant blood tests. - Explain follow-up support to be provided.   Materials:   - Prompt cards with ideas for healthy eating (smart eating), activity ideas (introducing FITT principles); infant and family nutrition; and strategies for being active with a young infant.   Targets:   - Patient remains motivated and activated. - Patient sets goals for 3-6 month period. - Patient is using the pedometer and WhatsApp group. |
| **Session 4: 6 months postpartum (60 minutes)** |
| Content:   - Reflect on their previous goals and experiences to see what can be learned and support with affirmational feedback. - Elicit what the patient now thinks will be important to reduce their risk and provide menu of potential actions that may reduce risk- explore with patient. - Explore importance and confidence of action areas- develop self-efficacy as required. - If patient is ready progress to setting lifestyle goals (diet and physical activity) with patient using SMART model; if patient is not ready explore further pros and cons of status quo and potential for change with reflective exercises. - Review pedometer use and explore with patient strategies to increase steps- set a new target. - Review use of WhatsApp group. - Explain follow-up support to be provided. - Discuss weight and any relevant blood tests.   Materials:   - Prompt-card with activity strategies reflecting needs of an older infant (6-12 months). - Prompt- card exploring health eating strategies for the infant and whole family.   Targets:   - Patient remains motivated and activated. - Patient identifies resources and strategies that will help them fulfil goals. - Patient sets goals or reflective exercises for 6-9 month period. - Patient is using the pedometer and WhatsApp group. |
| **Session 5: Maintenance Session 9 months postpartum (40 mins)** |
| Content:   - Reflect on their previous goals, experiences and what they have achieved to see what can be learned and support with affirmational feedback. - Elicit what the patient now thinks will be important to in helping them to extend and or maintain their lifestyle changes. - Encourage patient to identify longer term goals and strategies to enable them to maintain a healthy lifestyle for themselves, their infant and their family. - Elicit from patient the strategies they could use to help them know they were remaining on track with their plan. - Review pedometer use and explore with patient if they want to continue using the pedometer to help with their maintenance. - Review use of self-help resources and introduce and discuss with patient how they can use these resources to help them maintain a healthy lifestyle. - Explain follow-up support to be provided. - Discuss weight and any relevant blood tests.   Targets:   - Patient has identified strategies to help them maintain their health changes, together with indicators for recognising if they may be relapsing. - Patient will continue to use the pedometer and WhatsApp group. |

Alongside these sessions, participants will be provided with access to a facilitated ‘WhatsApp’ group, in order to connect with other participants in the trial, allow the opportunity for discussion and peer support.

**4.5 Follow up Procedures**

At the end of the study participants randomised to the usual care group will be given an opportunity to ask questions and receive information.

**4.6 End of Study Definition**

Once the primary outcome variables have been appropriately assessed and data analysis completed, this marks the end of the study.

### 5. Laboratories

All blood samples will be analysed in the Viapath laboratory using quality assured procedures.

**5.1 Central/Local Laboratories**

Blood Samples will be collected locally and sent to the Viapath lab.

**5.2 Sample Collection/Labelling/Logging**

Blood samples will be tracked and identifiable by the unique patient code. Sample results will be entered onto the patient record.

**5.3 Sample analysis procedure**

The sample analysis will be done through Viapath

**5.4 Sample storage procedure**

Sample will be destroyed

**5.5 Data Recording/Reporting**

The study will be written up as a thesis for a Doctorate Degree. The results will be reported in peer reviewed scientific journals, presented at relevant conferences as a poster or presentations such as Diabetes UK conference, Foundation of European Nurses (FEND) conference, Diabetes, Hypertension, Metabolic Syndrome and Pregnancy symposium (DIP), and others.

We will convene a patient seminar at the end of the study inviting all participants and other patient organisations to attend and provide comment and feedback on the study findings

### 6. Assessment of Safety

We have carefully considered the potential clinical hazards associated with this study. These have been discussed by clinical colleagues not directly involved in the study. There are no major risks involved with participation in this study. The study will have in place the following safety monitoring procedures: an incident log for every patient to record any adverse events; an incident log for all clinical research areas to monitor adherence to study protocol and SOP and for recording any incidents that may occur in the conduct of the study; weekly team meetings to consider any difficulties or potential hazards; and clear line of reporting for any adverse events. All team members have completed the KHP Good Clinical Practice. The clinical team will be advised of the patient’s participation in the study to ensure no contradicting intervention during the study.

The responsibility for the conduct of the study resides with all team members with overall responsibility resting with the Chief Investigator (CI). In addition to the weekly team meetings a monthly trial monitoring committee will be held for the duration of the study. This committee is comprised of: the CI, motivational interviewer, 1 diabetes consultant psychiatrist, 1 diabetes medical consultant, 1 diabetes nurse consultant, 2 researchers, a patient representative and a representative from the CTU.

Ethical issues around provision of information to the usual care group will be addressed by providing patients in this group with information about diabetes and healthy lifestyles after completion of follow-up measurements.

**6.1 Ethics Reporting**

We do not consider that there are any major ethical dilemmas in relation to this study. All patients will give fully informed written consent for their participation, which will be gained from an appropriately trained medical professional. The study will proceed subject to NHS Research Ethics Committee approval and Health Research Authority approval. The study will be indemnified by the no-fault insurance policy of King’s College London.

### 7. Trial Steering Committee

We will have a study committee comprised of: the CI, PIs, 1 diabetes consultant psychiatrist, 1 diabetes nurse consultant, 1 researcher and a patient representative.

### 8. Ethics & Regulatory Approvals

The study will be reviewed by ……..Ethics

### 9. Data Handling

**9.1 Confidentiality**

Eligibility Checklist Forms and the Participant Information and signed Consent Forms will be printed, signed and dated by the research team and a copy will be returned to the participant and another copy to the hospital medical notes.

The printed forms will be stored within a secure lockable file location at King’s College London. In addition, a copy of each of these forms for each participant are stored electronically within King’s College’s secure research database.

All participants recruited to the study are recorded and tracked on the King’s college secure research project database. Participant tracking records include the participant’s personal details, when their eligibility and consent forms were received. These participant tracking records then form the base for participant tracking throughout the study, which highlights the allocated ID number for participants and the study visits and follow up dates for the duration of the study.

Participants will be reassured that the information they provide will be confidential. We will also ensure it is not possible to identify participants from our publically available research reports.

**9.2 Case Report Form (CRF)**

Each participant within this study will be assessed to ensure that they have undergone all appropriate procedure and to monitor patient safety throughout the course of the study. The CRF incorporates the following:

- Eligibility/exclusion criteria checklist include: Demographic and biometric data, Insulin data, weight and BMI; and diabetes complications, history of DKA, current medicines and past-medical history (from the medical record).
- Blood test results (fasting blood glucose, HbA1c)
- weight, height, BMI, waist circumference
- Visit details
- Adverse events, withdrawal from study, SAE form
- Study questionnaires include: Multiple pass 24 hour diet recall, Beacke physical activity questionnaire, Risk Perception Survey for Developing Diabetes, Hospital anxiety and depression scale

**9.3 Record Retention and Archiving**

The Chief Investigator (CI) and the research team will have access to participants' personal data during the study. Participants will be informed, both verbally and through a Patient Information Sheet, that the CI and the research team will access their medical records during the study, in order to record their clinical information. Participants will sign an Informed Consent to confirm their willingness for the research team to access their personal information*.*

Data files will be stored on a password protected computer at the researcher’s University. Paper questionnaires will be stored in a locked filing cabinet in a research office at King's College London (KCL). This data will be primarily analysed by the PhD student with discussion of the analysis with academic supervisors and members of the research team throughout. Support from a statistician will be sought in case of need. Data analysis will be carried out in KCL, using SPSS on a password protected university computer.

Each participant will be assigned a unique study identifier and all data will be coded with that study identifier. Personalised information with the unique study identifier will be managed securely in restricted access, lockable containers at King’s College London (KCL).

No personalised data will be stored electronically, all data will be anonymised and any electronic data will be stored on a password protected university computers. The data held on these computers will not be identifiable back to personalised data.

Following completion of the study the personal data will be stored locally and accessed for 6-12 months after the study has ended. The electronic data generated by the study will be stored electronically on a university computer at King’s College London for 5 years. It will not contain any participant identifiable data and will only be used if there are any queries regarding the study after publication and future study design.

**9.4 Compliance**

The CI will ensure that the trial is conducted in compliance with the principles of the Declaration of Helsinki (1996), and in accordance with all applicable regulatory requirements including but not limited to the Research Governance Framework, Trust and Research Office policies and procedures and any subsequent amendments*.*

**9.5 Clinical Governance Issues**

The sponsor will determine the appropriate level and nature of monitoring required for the trial.  Risk will be assessed on an ongoing basis and adjustments made accordingly. The degree of monitoring will be proportionate to the risks associated with the trial. A trial specific oversight and monitoring plan will be established for studies. The trial will be monitored in accordance with the agreed plan

**9.6 Non-Compliance**

The sponsor will maintain a log of the non-compliances to ascertain if there are any trends developing which to be escalated. The sponsor will assess the non-compliances and action a timeframe in which they need to be dealt with. Each action will be given a different timeframe dependant on the severity. If the actions are not dealt with accordingly, the R&D Office will agree an appropriate action, including an on-site audit.

### 10. Finance and Publication Policy

**Amount: £241,208**

**Source of funding:** student bench fees from student sponsorship managed by KCL and KCH.

Name and address of funder

**Name:** NIHR Trainees Coordinating Centre

**Address:** Leeds Innovation Centre, 103 Clarendon Road, Leeds, LS2 9DF

**Telephone:** 0113 346 6260

**Email:** [tcc@nihr.ac.uk](mailto:tcc@nihr.ac.uk)

The trial will be registered on the ClinicalTrials.org. Part of the research will be written up as a thesis for a doctorate at King’s College London. The results will be disseminated locally, nationally and internationally in meetings and conferences for healthcare professionals. Papers will be published in health journals associated with diabetes and newsletters for people with diabetes.

**Appendix 1 – Information with regards to Safety Reporting in Non-CTIMP Research**

|  | **Who** | **When** | **How** | **To Whom** |
| --- | --- | --- | --- | --- |
| **SAE** | Chief Investigator | -Report to Sponsor within 24 hours of learning of the event  -Report to the MREC within 15 days of learning of the event | SAE Report form for Non-CTIMPs, available from NRES website. | Sponsor and MREC |
| **Urgent Safety Measures** | Chief Investigator | Contact the Sponsor and MREC Immediately  Within 3 days | By phone  Substantial amendment form giving notice in writing setting out the reasons for the urgent safety measures and the plan for future action. | Main REC and Sponsor  Main REC with a copy also sent to the sponsor. The MREC will acknowledge this within 30 days of receipt. |
| **Progress Reports** | Chief Investigator | Annually ( starting 12 months after the date of favourable opinion) | Annual Progress Report Form (non-CTIMPs) available from the NRES website | Main REC |
| **Declaration of the conclusion or early termination of the study** | Chief Investigator | Within 90 days (conclusion)  Within 15 days (early termination)  *The end of study should be defined in the protocol* | End of Study Declaration form available from the NRES website | Main REC with a copy to be sent to the sponsor |
| **Summary of final Report** | Chief Investigator | Within one year of conclusion of the Research | No Standard Format  However, the following Information should be included:-  Where the study has met its objectives, the main findings and arrangements for publication or dissemination including feedback to participants | Main REC with a copy to be sent to the sponsor |

**REFERENCES**

1. The, L., *The global challenge of diabetes.* The Lancet. **371**(9626): p. 1723.

2. Ferrara, A., *Increasing prevalence of gestational diabetes mellitus.* Diabetes care, 2007. **30**(Supplement 2): p. S141-S146.

3. Bellamy, L., et al., *Type 2 diabetes mellitus after gestational diabetes: a systematic review and meta-analysis.* The Lancet, 2009. **373**(9677): p. 1773-1779.

4. Hanna, F. and J. Peters, *Screening for gestational diabetes; past, present and future.* Diabetic medicine, 2002. **19**(5): p. 351-358.

5. Kim, C., D.K. Berger, and S. Chamany, *Recurrence of gestational diabetes mellitus.* Diabetes care, 2007. **30**(5): p. 1314-1319.

6. Dabelea, D., W.C. Knowler, and D.J. Pettitt, *Effect of diabetes in pregnancy on offspring: follow-up research in the Pima Indians.* Journal of maternal-fetal medicine, 2000. **9**(1): p. 83-88.

7. Catalano, P.M., et al., *The hyperglycemia and adverse pregnancy outcome study.* Diabetes care, 2012. **35**(4): p. 780-786.

8. UK, D., *Diabetes: facts and Stats*. 2016.

9. Group, D.P.P.R., *Reduction in the incidence of type 2 diabetes with lifestyle intervention or metformin.* N Engl j Med, 2002. **2002**(346): p. 393-403.

10. Tuomilehto, J., et al., *Prevention of type 2 diabetes mellitus by changes in lifestyle among subjects with impaired glucose tolerance.* New England Journal of Medicine, 2001. **344**(18): p. 1343-1350.

11. Ratner, R.E., et al., *Prevention of diabetes in women with a history of gestational diabetes: effects of metformin and lifestyle interventions.* The Journal of Clinical Endocrinology & Metabolism, 2008. **93**(12): p. 4774-4779.

12. Kim, C., et al., *A web‐based pedometer programme in women with a recent history of gestational diabetes.* Diabetic Medicine, 2012. **29**(2): p. 278-283.

13. McIntyre, H.D., et al., *Pilot study of an individualised early postpartum intervention to increase physical activity in women with previous gestational diabetes.* International journal of endocrinology, 2012. **2012**.

14. Ferrara, A., et al., *A pregnancy and postpartum lifestyle intervention in women with gestational diabetes mellitus reduces diabetes risk factors: a feasibility randomized control trial.* 2011.

15. Cheung, N., et al., *A pilot structured behavioural intervention trial to increase physical activity among women with recent gestational diabetes.* Diabetes research and clinical practice, 2011. **92**(1): p. e27-e29.

16. Kim, C., K.M. Newton, and R.H. Knopp, *Gestational diabetes and the incidence of type 2 diabetes.* Diabetes care, 2002. **25**(10): p. 1862-1868.

17. Lindström, J., et al., *Take action to prevent diabetes–the IMAGE toolkit for the prevention of type 2 diabetes in Europe.* Hormone and Metabolic research, 2010. **42**(S 01): p. S37-S55.

18. Graco, M., J. Garrard, and A.E. Jasper, *Participation in physical activity: perceptions of women with a previous history of gestational diabetes mellitus.* Health Promotion Journal of Australia, 2009. **20**(1): p. 20-25.

19. Nicklas, J.M., et al., *Identifying postpartum intervention approaches to prevent type 2 diabetes in women with a history of gestational diabetes.* BMC Pregnancy and Childbirth, 2011. **11**(1): p. 23.

20. Parsons, J., et al., *Perceptions among women with gestational diabetes.* Qualitative health research, 2014. **24**(4): p. 575-585.

21. Briley, A.L., et al., *A complex intervention to improve pregnancy outcome in obese women; the UPBEAT randomised controlled trial.* BMC pregnancy and childbirth, 2014. **14**(1): p. 74.

22. Poston, L., et al., *Effect of a behavioural intervention in obese pregnant women (the UPBEAT study): a multicentre, randomised controlled trial.* The lancet Diabetes & endocrinology, 2015. **3**(10): p. 767-777.

23. Avalos, L., T.L. Tylka, and N. Wood-Barcalow, *The body appreciation scale: Development and psychometric evaluation.* Body image, 2005. **2**(3): p. 285-297.

24. Karlsson, J., et al., *Psychometric properties and factor structure of the Three-Factor Eating Questionnaire (TFEQ) in obese men and women. Results from the Swedish Obese Subjects (SOS) study.* International journal of obesity, 2000. **24**(12): p. 1715.

25. Satia, J.A., et al., *Motivations for healthful dietary change.* Public Health Nutrition, 2001. **4**(5): p. 953-959.
